# Ocean weather, biological rates, and unexplained global ecological patterns

**DOI:** 10.1093/pnasnexus/pgae260

**Published:** 2024-08-06

**Authors:** Darren L C Y Li Shing Hiung, Jasmin M Schuster, Murray I Duncan, Nicholas L Payne, Brian Helmuth, Jackson W F Chu, Julia K Baum, Viviana Brambilla, John Bruno, Sarah W Davies, Maria Dornelas, Patrick Gagnon, Tamar Guy-Haim, Jennifer M Jackson, James J Leichter, Joshua S Madin, Zachary L Monteith, Ana M Queirós, Eric V C Schneider, Samuel Starko, Brendan S Talwar, Alex S J Wyatt, Hannah E Aichelman, Nathaniel Bensoussan, Carlo Caruso, Karl Castillo, Francis Choi, Yun-Wei Dong, Joaquim Garrabou, Dorian Guillemain, Nicholas Higgs, Yuwu Jiang, Diego K Kersting, David J Kushner, Guilherme O Longo, Christopher Neufeld, Marion Peirache, Tim Smyth, Joshua L Sprague, Gaëlle Urvoy, Frederic Zuberer, Amanda E Bates

**Affiliations:** Department of Ocean Sciences, Memorial University of Newfoundland, 0 Marine Lab Road, St. John’s, NL, Canada A1C 5S7; Oceans and Cryosphere Centre, Institute for Marine and Antarctic Studies, University of Tasmania, 20 Castray Esplanade, Battery Point, TAS 7004, Australia; Department of Ocean Sciences, Memorial University of Newfoundland, 0 Marine Lab Road, St. John’s, NL, Canada A1C 5S7; Biology Department, University of Victoria, 3800 Finnerty Road, Victoria, BC, Canada V8P 5C2; Department of Environment, University of Seychelles, 7G58+R5R, Anse Royale, Mahe, Seychelles 0000; Blue Economy Research Institute, University of Seychelles, 7G58+R5R, Anse Royale, Mahe, Seychelles 0000; Department of Ichthyology and Fisheries Science, Rhodes University, Prince Alfred St, Grahamstown, Makhanda, 6139, South Africa; Department of Zoology, School of Natural Sciences, Trinity College Dublin, College Green, Dublin 2, D02 PN40, Ireland; Department of Marine and Environmental Sciences, Northeastern University, 360 Huntington Ave, Boston, MA 02115, USA; St. Andrews Biological Station, Fisheries and Oceans Canada, 125 Marine Science Drive, St. Andrews, New Brunswick, Canada E5B OE4; Biology Department, University of Victoria, 3800 Finnerty Road, Victoria, BC, Canada V8P 5C2; Centre for Biological Diversity, School of Biology, University of St Andrews, Dyers Brae House, Greenside Place, St Andrews KY16 9TH, United Kingdom; MARE—Centro de Ciências do Mar e do Ambiente, Faculdade de Ciências, Universidade de Lisboa, Av. Nossa Senhora do Cabo, 939, 2750-374 Cascais, Portugal; Department of Biology, The University of North Carolina at Chapel Hill, 120 South Rd, Chapel Hill, NC 27599, USA; Department of Biology, Boston University, 5 Cummington Mall, Boston, MA 02215, USA; Centre for Biological Diversity, School of Biology, University of St Andrews, Dyers Brae House, Greenside Place, St Andrews KY16 9TH, United Kingdom; MARE—Centro de Ciências do Mar e do Ambiente, Faculdade de Ciências, Universidade de Lisboa, Av. Nossa Senhora do Cabo, 939, 2750-374 Cascais, Portugal; Department of Ocean Sciences, Memorial University of Newfoundland, 0 Marine Lab Road, St. John’s, NL, Canada A1C 5S7; Marine Biology Department, National Institute of Oceanography, Israel Oceanographic and Limnological Research, Tel-Shikmona, Haifa, Israel; Hakai Institute, 1703 Hyacinthe Bay Road, Heriot Bay, BC, Canada V0P 1N0; Institute of Ocean Sciences, Fisheries and Oceans Canada, 9860 W Saanich Rd, Sidney BC, Canada V8L 4B2; Scripps Institution of Oceanography, University of California San Diego, 8622 Kennel Way, La Jolla, CA 92093, USA; Hawai‘i Institute of Marine Biology, University of Hawai‘i at Mānoa, 46-007 Lilipuna Road, Kāne‘ohe, HI 96744, USA; Hakai Institute, 1703 Hyacinthe Bay Road, Heriot Bay, BC, Canada V0P 1N0; Plymouth Marine Laboratory, Prospect Place, Plymouth PL1 3DH, United Kingdom; Cape Eleuthera Institute, Cape Eleuthera Island School, PO Box EL-26029, Rock Sound, Eleuthera, The Bahamas; Biology Department, University of Victoria, 3800 Finnerty Road, Victoria, BC, Canada V8P 5C2; Oceans Institute & School of Biological Sciences, University of Western Australia, 54 Fairway, Crawley, WA 6009, Australia; Cape Eleuthera Institute, Cape Eleuthera Island School, PO Box EL-26029, Rock Sound, Eleuthera, The Bahamas; Scripps Institution of Oceanography, University of California San Diego, 8622 Kennel Way, La Jolla, CA 92093, USA; Department of Ocean Science, The Hong Kong University of Science and Technology, Clear Water Bay Rd, Kowloon, Hong Kong 0000; Department of Biology, Boston University, 5 Cummington Mall, Boston, MA 02215, USA; Aix Marseille Univ, Université de Toulon, CNRS, IRD, Institut Méditerranéen d’Océanologie (MIO), Campus de Luminy, Case 901, Océanomed, Bât. Méditerranée 26M/102, 13288 Marseille Cédex 9, France; Hawai‘i Institute of Marine Biology, University of Hawai‘i at Mānoa, 46-007 Lilipuna Road, Kāne‘ohe, HI 96744, USA; Department of Earth, Marine and Environmental Sciences, University of North Carolina at Chapel Hill, Mitchell Hall, 104 South Rd, Chapel Hill, NC 27514, USA; Department of Marine and Environmental Sciences, Northeastern University, 360 Huntington Ave, Boston, MA 02115, USA; Key Laboratory of Mariculture of Ministry of Education, Fisheries College, Ocean University of China, 5 Yushan Road, Qingdao, 266001, China; Departament de Biologia Marina, Institut de Ciències del Mar-CSIC, Passeig Marítim de la Barceloneta, 37, Ciutat Vella, 08003 Barcelona, Spain; Aix-Marseille Université, CNRS, IRD, IRSTEA, OSU-Pytheas, 163 Avenue de Luminy—Bâtiment Oceanomed, 13288 Marseille Cedex 09, France; Cape Eleuthera Institute, Cape Eleuthera Island School, PO Box EL-26029, Rock Sound, Eleuthera, The Bahamas; Department of Physical Oceanography, National Observation and Research Station for the Taiwan Strait Marine Ecosystem, Xiamen University, 422 Siming S Rd, Siming District, Xiamen, Fujian, 361011, China; Instituto de Acuicultura Torre de la Sal (IATS), CSIC, 12595 Ribera de Cabanes, Castelló, Spain; Division of Natural Resources Management, Channel Islands National Park, 1901 Spinnaker Dr., Ventura, CA 93001, USA; Marine Ecology Laboratory, Department of Oceanography and Limnology, Universidade Federal do Rio Grande do Norte, Av. Via Costeira Senador Dinarte Medeiros Mariz, S/N, Natal, RN, 59014-002, Brazil; Department of Biology, University of British Columbia Okanagan, 1177 Research Road, Kelowna, BC, Canada V1V 1V7; Parc National de Port-Cros, 181 Allée du Castel Sainte-Claire, 83406 Hyères Cedex, France; Plymouth Marine Laboratory, Prospect Place, Plymouth PL1 3DH, United Kingdom; Division of Natural Resources Management, Channel Islands National Park, 1901 Spinnaker Dr., Ventura, CA 93001, USA; Parc National de Port-Cros, 181 Allée du Castel Sainte-Claire, 83406 Hyères Cedex, France; PSL Université Paris: EPHE-UPVD-CNRS, UAR 3278 CRIOBE, Université de Perpignan, 52 Avenue Paul Alduy, 66860 Perpignan Cedex, France; Department of Ocean Sciences, Memorial University of Newfoundland, 0 Marine Lab Road, St. John’s, NL, Canada A1C 5S7; Biology Department, University of Victoria, 3800 Finnerty Road, Victoria, BC, Canada V8P 5C2

**Keywords:** in situ, ocean temperature, high frequency, biological rate, climate variability hypothesis

## Abstract

As on land, oceans exhibit high temporal and spatial temperature variation. This “ocean weather” contributes to the physiological and ecological processes that ultimately determine the patterns of species distribution and abundance, yet is often unrecognized, especially in tropical oceans. Here, we tested the paradigm of temperature stability in shallow waters (<12.5 m) across different zones of latitude. We collated hundreds of in situ, high temporal-frequency ocean temperature time series globally to produce an intuitive measure of temperature variability, ranging in scale from quarter-diurnal to annual time spans. To estimate organismal sensitivity of ectotherms (i.e. microbes, algae, and animals whose body temperatures depend upon ocean temperature), we computed the corresponding range of biological rates (such as metabolic rate or photosynthesis) for each time span, assuming an exponential relationship. We found that subtropical regions had the broadest temperature ranges at time spans equal to or shorter than a month, while temperate and tropical systems both exhibited narrow (i.e. stable) short-term temperature range estimates. However, temperature-dependent biological rates in tropical regions displayed greater ranges than in temperate systems. Hence, our results suggest that tropical ectotherms may be relatively more sensitive to short-term thermal variability. We also highlight previously unexplained macroecological patterns that may be underpinned by short-term temperature variability.

**Significance Statement**

We collated hundreds of temperature time series from around the world's oceans recorded at a frequency of 1 hour or less. Using these data, we tested for patterns in temperature variability across climate regions. Contrary to the climate variability hypothesis, which states that the temperature variability is highest in temperate regions and lowest in tropical ones, our results show that, in the short term, subtropical regions tend to be most variable. To investigate the biological significance of this pattern, we converted our measure of temperature variability into the equivalent span of biological rates that would be experienced by an ectothermic organism at equilibrium with its environment. Our findings could help to explain ecological patterns that were previously unexplained.

## Introduction

Recording ocean temperature over large spatial scales (e.g. 1,000 s km) and continuously through time at scales relevant to the body temperatures of microbes, algae, and other marine animals has historically been challenging simply because the ocean is so vast (1). Since the 1980s, it has become more feasible to measure the global ocean temperature at the sea surface using infrared sensors aboard satellites (2). As a result, sea surface temperature (SST) has been used in many studies as a proxy for in situ temperature in the oceans [e.g. (3)]. Yet, SST data are often averaged over large spatial (e.g. from multiple km^2^ up to 1-by-1 degree grids) and temporal (e.g. daily data for once-per-day satellite passes) scales, which can mask finer-scale variability that could otherwise be captured using high frequency in situ temperature loggers (4). For instance, satellite-derived time series, aggregating temperature data at much coarser spatial scales, are frequently and typically employed to calibrate Earth System Model projections (5). The latter are then used to drive ecological niche models that are often used to forecast climate-driven changes in species distributions (6). While the remotely sensed data used at the basis of these applications are typically calibrated using fine-scale in situ data [e.g. (7)], because these remotely sensed estimates of SST have a much coarser resolution, the finer-scale variability experienced by individual organisms is not captured in those estimations.

In the oceans, fine-scale temperature variability can arise due to oceanographic processes, such as upwelling, tides, and eddies, and is known to drive many ecological patterns (8–12) including mass mortality events during and following marine heatwaves and cold-spells (13–15) as well as the persistence and movements of organisms (16, 17). When local conditions exceed organismal thresholds, biodiversity losses can be dire (18, 19). Ultimately, records that include local-scale temperature variability may be critical to improving understanding of processes that drive the physiological performance, reproduction and survival of organisms, and the dynamics of populations and assemblages (4, 18, 19). However, while in situ records of high spatial- and temporal-frequency ocean temperature data are widespread, their integrated use at macroecological scales is comparatively rare and has not yet been examined for ocean-wide comparisons across regions.

**Fig. 1.**
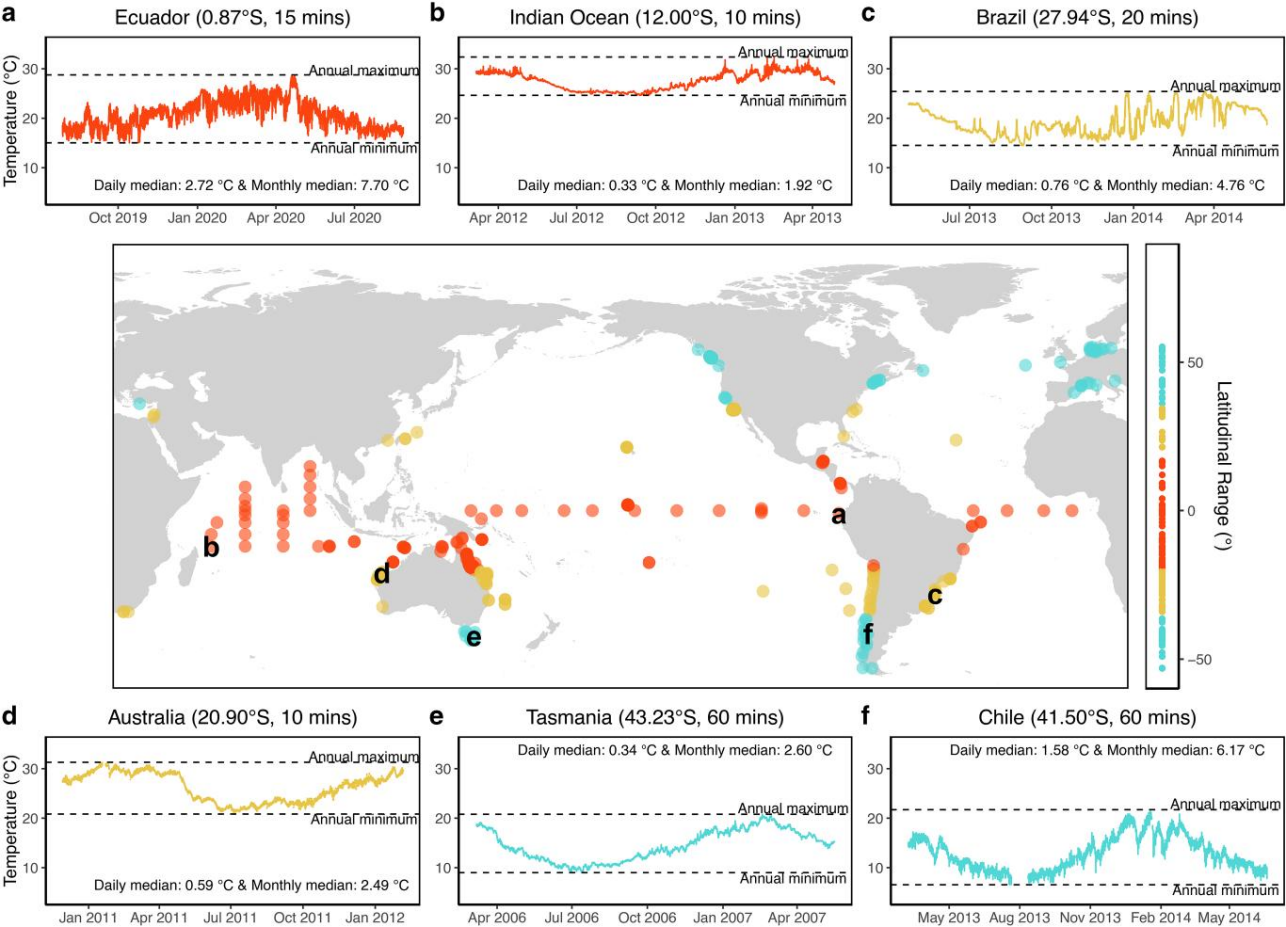
Distribution of the 492 high frequency temperature time series used in this study. 169 time series are from tropical regions, 179 from subtropical, and 144 from temperate regions. Insets a)–f) correspond to the temperature time series at the locations shown by the respective letters. These sample time series help to visualize the higher temporal variability of certain locations at the shorter temporal windows. For instance, insets a) and f) both show very high temperature variability [a) tropical Ecuador (2.72°C diurnal median, 7.70°C monthly median) and f) temperate Chile (1.58°C diurnal median, 6.17°C monthly median)], even though inset a) is from tropical regions. The numbers between brackets in the title of each inset indicate the latitude and the measurement frequency of the respective time series. The median temperature ranges over the diurnal and monthly temporal windows are also shown for each inset. The *y*-axes of all six insets have the same range, for comparability. Additional examples of these time series are shown in SI Appendix, Figs. S6 to S8.

Here, we investigated the “tropical temperature stability paradigm” (20, 21) at short timescales by testing whether shallow (<12.5 m) tropical ocean temperatures are more stable in comparison with locations from higher latitudes at time spans of less than a year. To do so, we first assembled 492 in situ ocean temperature time series measured at high temporal resolutions for tropical, subtropical, and temperate locations across a wide range of latitude (Fig. 1). We then used these records to quantify temperature variability at different temporal windows (quarter-diurnal, semi-diurnal, diurnal, weekly, bi-weekly, monthly, and annual) based on common astronomical cycles and biorhythms by calculating the range of temperatures (difference between the minimum and maximum) for each temporal window.

Most marine species are ectotherms (22) whose biological processes are dependent on temperature (e.g. metabolic rate and photosynthesis). Thus, temperature variability presumably plays an important role across all levels of biological organization. To examine the potential biological impact of the measured temperature variability, we also modeled biological processes that track temperature using metabolic rate as an example. More specifically, we used a biological rate equation that assumes an exponential relationship with temperature (23, 24) within the rise portion of the thermal performance curve. Our approach moves beyond more typical efforts that assess the relative sensitivity of species living at the edge of their thermal safety margins (TSMs) [e.g. (25)] or when rates (e.g. photosynthesis) saturate and ultimately fall due to limiting factors such as light, nutrient, and carbon availability (26). Thus, our results apply only to the rise component of temperature-dependent biological processes and consider sensitivity to temperature variability within this specific range.

## Results

### Ocean temperature variability

**Fig. 2.**
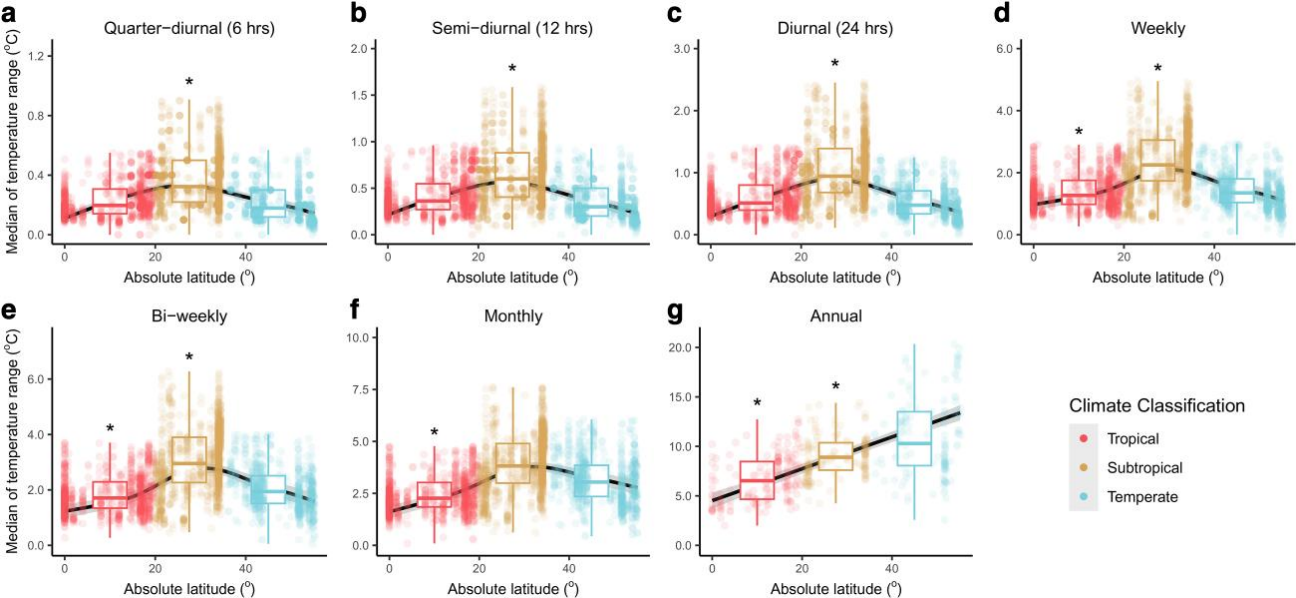
Median of the temperature range over seven different temporal windows as a function of the absolute latitude. The median temperature range for seven temporal windows: quarter-diurnal (a, *n* = 3,063), semi-diurnal (b, *n* = 3,066), diurnal (c, *n* = 3,035), weekly (d, *n* = 3,034), bi-weekly (e, *n* = 3,038), monthly (f, *n* = 3,035), and annual (g, *n* = 479). Dots represent the computed median temperature ranges over their respective temporal windows and are colored according to three climate classifications. The trends across absolute latitude are visualized using GAMM, as represented by the black lines, with the gray shadings representing the 95% CIs. Asterisks indicate that the Bayesian models showed strong evidence (i.e. the 0.95 credible intervals do not include zero) that tropical and/or subtropical regions differed from temperate regions. In both the GAMM and Bayesian models, “plot_id” was nested within “spatial_blocks” for all temporal windows (a–f) except for the annual time span whereby “spatial_blocks” was the only random effect specified (g) (see Materials and methods for more details). The boxplots show the medians (thick central lines) and the quartiles of the data binned under each climate region. Note that the *y*-axes of the boxes have different ranges. Sensitivity tests were performed to ensure that the results are robust (see SI Appendix, Figs. S1 to S3 and S5).

**Fig. 3.**
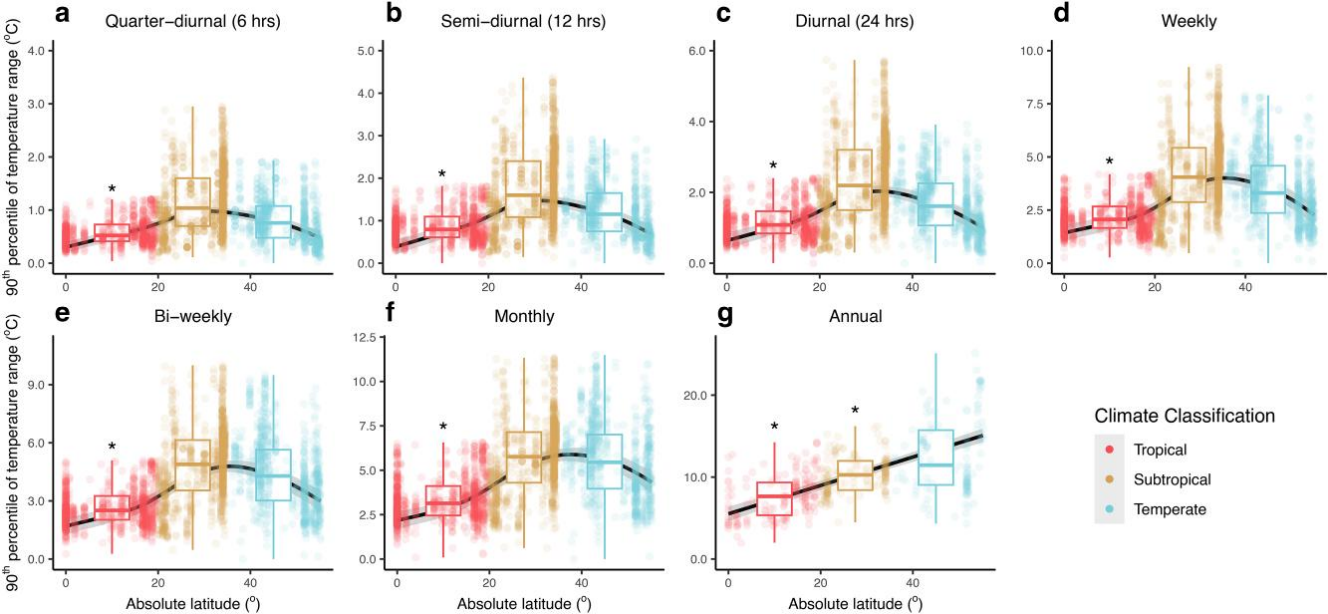
90th percentile of the temperature range over seven different temporal windows as a function of the absolute latitude. The extreme temperature range for seven temporal windows: quarter-diurnal (a, *n* = 3,044), semi-diurnal (b, *n* = 3,067), diurnal (c, *n* = 3,050), weekly (d, *n* = 3,050), bi-weekly (e, *n* = 3,045), monthly (f, *n* = 3,077), and annual (g, *n* = 482). Dots represent the computed extreme temperature ranges over their respective temporal windows and are colored according to three climate classifications. The trends across absolute latitude are visualized using GAMM, as represented by the black lines, with the gray shadings representing the 95% CIs. Asterisks indicate that the Bayesian models showed strong evidence (i.e. the 0.95 credible intervals do not include zero) that tropical and/or subtropical regions differed from temperate regions. In both the GAMM and Bayesian models, “plot_id” was nested within “spatial_blocks” for all temporal windows (a–f) except for the annual time span whereby “spatial_blocks” was the only random effect specified (g) (see Materials and methods for more details). The boxplots show the medians (thick central lines) and the quartiles of the data binned under each climate region. Note that the *y*-axes of the boxes have different ranges. Sensitivity tests were performed to ensure that the results are robust (see SI Appendix, Figs. S1 to S3 and S5).

The paradigm that the ocean temperature is most stable in the tropics was found to be true only at coarse temporal scales, i.e. annually. Indeed, our results revealed annual patterns of temperature variability in line with the tropical temperature stability paradigm, whereby both the median and 90th percentile of the temperature range for annual temporal windows (Figs. 2g and 3g) were highest in temperate systems and most stable in the tropics. The annual subtropical signal was intermediate between the temperature ranges in tropical and temperate regions.

However, our results for the short-term temperature variability showed a different trend across latitude which contradicts the “tropical temperature stability paradigm.” We found that the temperature ranges in the shorter temporal windows (quarter-diurnal to monthly) observed in the tropics could exceed those of temperate systems and did not support a paradigm of relative temperature stability in the tropics, at least at short time spans (see examples in Fig. 1). The median temperature range in the tropics was higher than that of temperate systems for the quarter-diurnal, semi-diurnal, and diurnal time spans (apparent in the generalized additive mixed-effects models (GAMM) and supported by Bayesian models, albeit weakly—see SI Appendix, Fig. S10) but was similar to that of temperate regions for the weekly and bi-weekly time spans (red versus blue boxes: Figs. 2a to 2e). The extreme (90th percentile) temperature ranges between tropical and temperate systems were also similar at quarter-diurnal, semi-diurnal, and diurnal time spans, whereby temperate systems only showed markedly higher extreme temperature ranges at longer time spans of weeks and above (red versus blue boxes: Figs. 3a to 3f). Our sensitivity analyses also supported similar trends (see SI Appendix, Figs. S1, S2, S3, and S5).

In general, the median temperature ranges in subtropical systems were highest at all short timescales as visualized by smoothed plots of the summed effects of GAMM across latitude (Fig. 2a to f). However, these reported trends for the monthly temporal window were not supported by the Bayesian mixed-effects model which included climate classification as a factor (as indicated by the absence of asterisks). Despite this, these trends were present in all our sensitivity tests (see SI Appendix, Figs. S1, S2, S3, and S5). The extreme (90th percentile) temperature range observations were also highest in subtropical regions at all short timescales, but temperate and subtropical regions became increasingly more similar as the time span increased from quarter-diurnal to monthly (yellow boxes: Fig. 3a to f). Moreover, when compared with the median temperature ranges (Fig. 2a to f), none of the Bayesian models for the extreme temperature ranges at short timescales showed strong evidence that the temperature variability in subtropical regions was highest (Fig. 3a to f), although the GAMM did show that these trends were still present. Here again, our sensitivity tests corroborated these patterns, with the exception that the variability in temperate regions overtook that of subtropical regions for the monthly temporal window in some cases (see SI Appendix, Figs. S1, S2, and S3).

### Range of biological rates

**Fig. 4.**
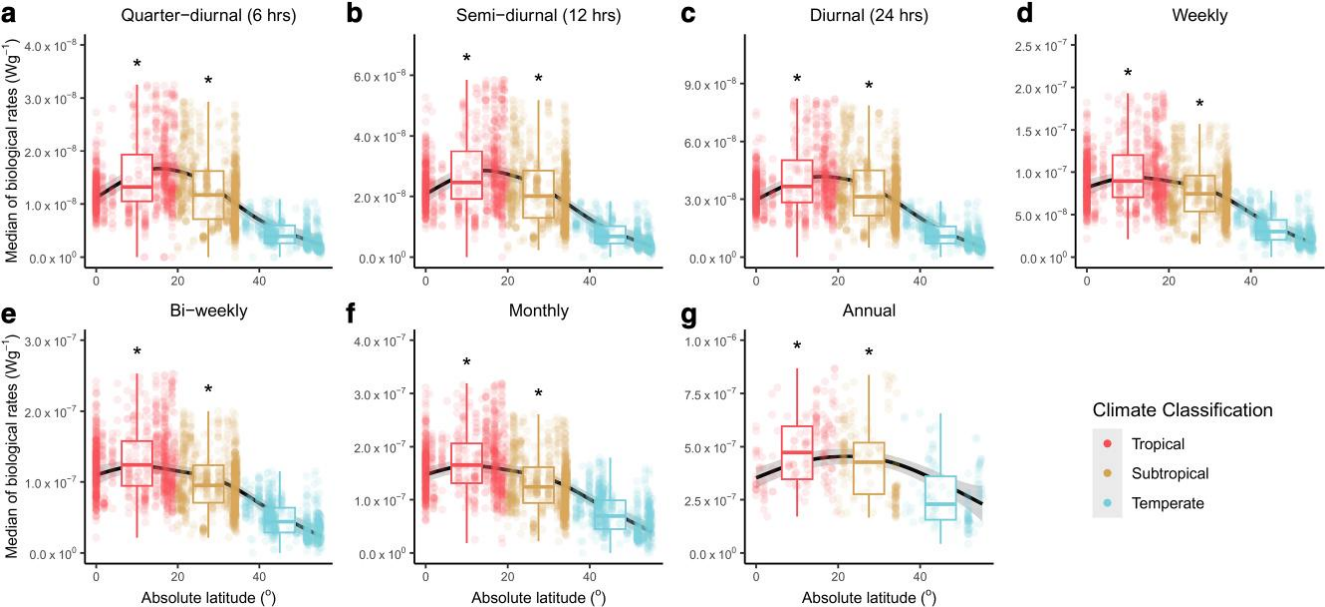
Median of the range of biological rates over seven different temporal windows as a function of the absolute latitude. The median of the range of biological rates for seven temporal windows: quarter-diurnal (a, *n* = 2,942), semi-diurnal (b, *n* = 2,961), diurnal (c, *n* = 2,932), weekly (d, *n* = 3,013), bi-weekly (e, *n* = 3,013), monthly (f, *n* = 2,984), and annual (g, *n* = 487). Dots represent the computed median of the range of biological rates over their respective temporal windows and are colored according to three climate classifications. The trends across absolute latitude are visualized using GAMM), as represented by the black lines, with the gray shadings representing the 95% CIs. Asterisks indicate that the Bayesian models showed strong evidence (i.e. the 0.95 credible intervals do not include zero) that tropical and/or subtropical regions differed from temperate regions. In both the GAMM and Bayesian models, “plot_id” was nested within “spatial_blocks” for all temporal windows (a–f) except for the annual time span whereby “spatial_blocks” was the only random effect specified (g) (see Materials and methods for more details). The boxplots show the medians (thick central lines) and the quartiles of the data binned under each climate region. Note that the *y*-axes of the boxes have different ranges. Sensitivity tests were performed to ensure that the results are robust (see SI Appendix, Figs. S1 to S5).

**Fig. 5.**
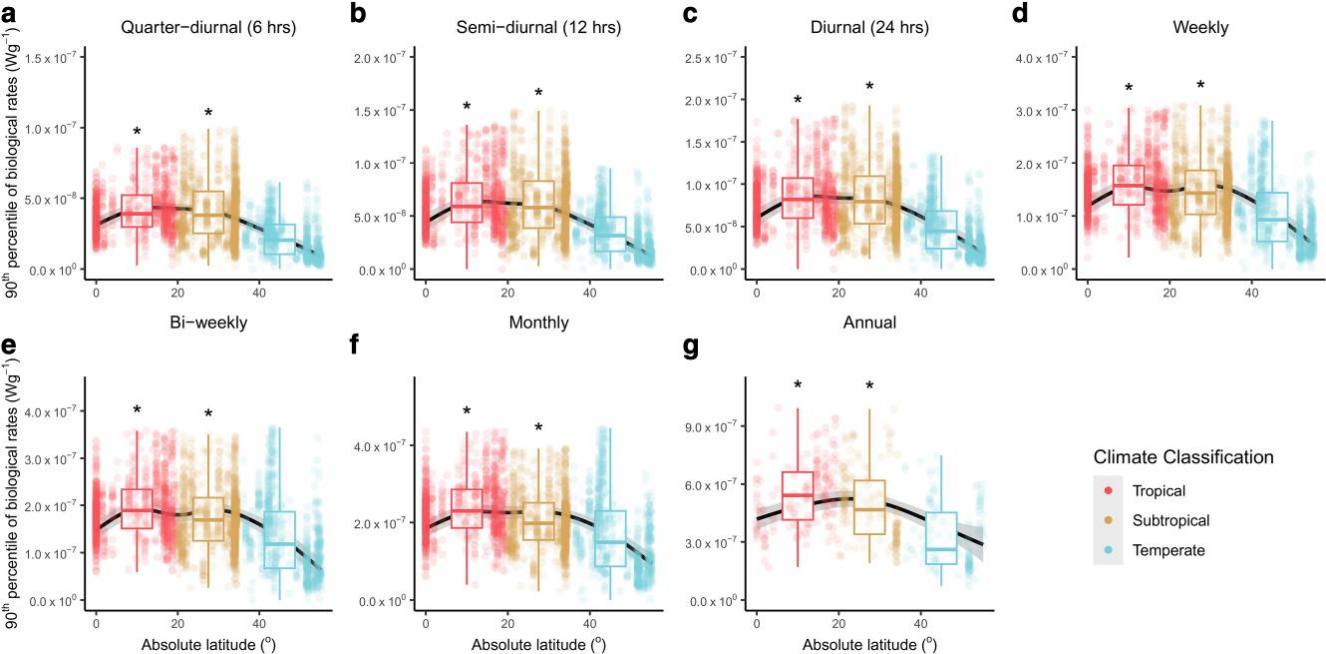
90th percentile of the range of biological rates over seven different temporal windows as a function of the absolute latitude. The extreme range of biological rates for seven temporal windows: quarter-diurnal (a, *n* = 3,035), semi-diurnal (b, *n* = 3,071), diurnal (c, *n* = 3,040), weekly (d, *n* = 3,063), bi-weekly (e, *n* = 3,043), monthly (f, *n* = 3,019), and annual (g, *n* = 488). Dots represent the computed extreme ranges of biological rates over their respective temporal windows and are colored according to three climate classifications. The trends across absolute latitude are visualized using GAMM, as represented by the black lines, with the gray shadings representing the 95% CIs. Asterisks indicate that the Bayesian models showed strong evidence (i.e. the 0.95 credible intervals do not include zero) that tropical and/or subtropical regions differed from temperate regions. In both the GAMM and Bayesian models, “plot_id” was nested within “spatial_blocks” for all temporal windows (a–f) except for the annual time span whereby “spatial_blocks” was the only random effect specified (g) (see Materials and methods for more details). The boxplots show the medians (thick central lines) and the quartiles of the data binned under each climate region. Note that the *y*-axes of the boxes have different ranges. Sensitivity tests were performed to ensure that the results are robust (see SI Appendix, Figs. S1 to S5).

We further found that converting these temperature ranges to the ranges of biological rates (i.e. the difference in $R_{o}e^{-\frac{E}{kT}}$ when *T* is substituted with the highest and lowest temperatures; see Materials and methods), led to different interpretations of “stability” across temperate, subtropical, and tropical regions. The range in temperature-dependent biological rates for temperate systems was consistently lower than that for both tropical and subtropical systems at both short and long time spans (Figs. 4 and 5), being typically highest for tropical systems. Although these reported trends are consistent in all our sensitivity tests for the median difference in biological rates, the trends are less consistent for the extreme difference in biological rates (see Fig. 5 and SI Appendix, Figs. S2, S3, and S4), but these small discrepancies do not affect the main patterns detected or our conclusions.

### Potential limitations

Our results certainly could have been affected by sampling bias, for instance, because many studies in the tropics aim to investigate internal waves [e.g. (27–29)] and thus select locations for deploying in situ temperature loggers that are inherently variable. However, our results are still likely robust because the 169 temperature time series from tropical regions used in this study come from a wide range of sources (see SI Appendix, Table S29), each with different goals. More specifically, the databases that contributed 61.5% of the time series in tropical regions [Australian Institute for Marine Science (AIMS): 66 time series, and Pacific Marine Environmental Laboratory (PMEL): 38 time series] are underpinned by more generic ocean monitoring objectives.

Our results could also have been affected by measurement uncertainties of the temperature sensors. Indeed, a low accuracy and/or resolution of the sensors relative to the temperature variability quantified in this study would result in a large uncertainty of the temperature variability. Here, we compiled in situ temperature data globally that were recorded with an array of different sensors having different accuracies and resolutions (see SI Appendix, Table S30). The sensor accuracies ranged from 0.002 to 0.8°C, while the resolutions ranged from 0.0001 to 0.14°C. While it is possible that the data recorded using the sensor with the lowest accuracy (Sensus Ultra loggers: 0.8°C) could have affected our results, since this accuracy is comparable to the temperature variability for the shorter temporal windows (quarter-diurnal, semi-diurnal, and diurnal—see Fig. 2), it is unlikely that these data had much effect on our overall results, because the median and the 90th percentile of the temperature range estimates are computed over a larger number of replicates for the shorter temporal windows, thus canceling out the measurement uncertainties to some degree (see Materials and methods—Temperature range quantification). For instance, if the median is computed from 365 temperature range values for the diurnal temporal window over a year, it would be computed from 730 temperature range estimates for the semi-diurnal window and 1,460 estimates for the quarter-diurnal window. Thus, despite the higher potential for the measurement uncertainty of the loggers to affect the median and 90th percentile of the temperature range estimates when the temporal window is shorter, this issue is offset by the larger number of sample values that are obtained in a shorter temporal window. In addition, <25% of the temperature time series were recorded using the Sensus Ultra loggers (109 out of 492—see SI Appendix, Table S30). This number is also a conservative value, since the lower(st) accuracy and precision values are recorded in Table S30 in the cases where several sensors were used (see SI Appendix, Table S30). In addition to measurement uncertainties of the temperature loggers, one data provider (COSYNA) has also reported site- and seasonal-dependent effects (biofouling near the coast from spring to early autumn) that could have affected the data accuracy beyond the instruments’ factory specifications. However, sensor drifts of only up to 0.03°C were observed in this case (see SI Appendix, Table S30), which is much smaller than our reported temperature variability, even for the shortest temporal window.

Finally, there are some uncertainties regarding the computation of the biological rates, in particular with the *E* (i.e. activation energy) values used. Here, we used *E* values of 0.630 and 0.433 eV to represent the span of average activation energies of metabolic reactions for different animal divisions and for fish only, respectively (see Materials and methods for more information). Our sensitivity tests show that if the *E* values are similar across ecoregions for the same organism, then our patterns for the biological rates are mostly robust to different *E* values (see SI Appendix, Fig. S4). It is currently unclear whether *E* values vary with ecoregion for the same animal group. Thus, investigating whether *E* values vary in different ecoregions is an important future research avenue.

## Discussion

Long-held assumptions about the relationship between environmental temperature variability and patterns in a species’ physiological sensitivity underpin predictions of vulnerability to future change. For instance, the “tropical temperature stability paradigm” states that the shallow ocean temperature in tropical regions is less variable than that at higher latitudes, both within and across years, and over evolutionary timescales, due to climate stability in tropical regions (20, 21). Hence, tropical species are typically considered “thermal specialists” because their temperature regime is not generally expected to select for physiological flexibility (30). Tropical species also often live closer to their thermal limits with a narrow thermal safety compared to those from colder regions (30). Thus, the assumptions of greater historical environmental stability and the higher likelihood of exceeding temperature thresholds in tropical regions mean that tropical ectotherms are particularly sensitive not only to long-term ocean warming (30) but also to temperature variability signals, such as heatwaves (31, 32). Our results show that tropical regions typically exhibit lower seasonal variation in oceanic temperature and are more stable than temperate regions at annual time spans and stronger finer-scale temperature variation in tropical oceans is prevalent. Indeed, at the quarter-diurnal, semi-diurnal, and diurnal temporal windows, temperature fluctuations in tropical and temperate regions were roughly similar (and surprisingly can even be greater in some tropical regions). Moreover, subtropical systems were more variable than both temperate and tropical systems at all short timescales, a result that was unexpected. Our analyses were performed for shallow depths because the large-scale availability of in situ temperature data near the ocean's surface enables us to systematically test differences in temperature variability across ecoregions. Future research can look into global ocean temperature variation at greater depths when more data become available and test whether the same conclusions can be reached.

Here, we also investigated the potential biological effects of observed temperature variability by translating it to its corresponding biological rate to model biological processes, such as metabolic rate. The body temperature of ectotherms dictates temperature-dependent biological rates (23, 33) that respond through thermodynamic effects on enzyme kinetics (8, 34). In general, biological rates increase exponentially with environmental temperature below the point of physiological collapse (i.e. the optimal temperature, *T*_opt_) of the thermal performance curve (33). This happens as a result of faster cell kinetics leading to increased ATP demand and greater protein turnover (35), or as a result of higher oxygen demand to support higher metabolic rates including activity. Because of the exponential nature of the curve's rise component, a one-degree temperature increase in the tropics (e.g. from 24 to 25°C) will lead to greater changes in any temperature-dependent biological rates compared to a one-degree rise in colder regions (33) (e.g. from 9 to 10°C). Contrary to many studies that have focused on the consequences of the temperatures exceeding the TSMs of species in the tropics (temperatures that lead to a fall in biological rates), here, we focus on the realized response of a temperature-dependent rate change within the range of optimal environmental temperatures (i.e. over the rise component of the thermal performance curve) for a theoretical organism. Indeed, many other factors can affect the biological processes of marine species across latitude, such as solar radiation (36), nutrient supply (37), and water column mixing/stratification (37), and we hope to inspire future studies aiming to test how additional processes interplay with temperature variability to constrain biological patterns.

When the observed ocean temperature ranges were converted to the equivalent span of biological rates to model the rise portion of the thermal performance curve, the median span of biological rates was higher at all timescales in both the tropics and subtropics compared to that of temperate regions. Since the biological rates of ectotherms increase exponentially with temperature, ectotherms living in tropical and subtropical systems (i.e. at higher temperatures) may experience greater realized biological rate variability in comparison with those from temperate regions. This presumably comes with energy and efficiency consequences (38). In other words, the cost of living in warmer waters would presumably be relatively high (39) if short-term physiological acclimation needs to adjust reaction rates to track temperature change, even if the temperature variability in these warm waters is similar or lower than what is found in cooler locations. Our results therefore implicate the role of temperature fluctuations that fall within the TSMs as an additional “cost” for tropical species (40), which may ultimately explain why marine ectotherms from tropical regions tend to have a narrower TSM (23, 25).

Overall, detecting high frequency temperature signals across the shallow ocean begs the question of whether short-term in situ ocean temperature variability is important to species’ responses to warmer temperatures under climate change (17). In fact, three previously unexplained patterns from recent macroecological studies may be related to these short-term ocean temperature variability signals. First, “tropical” fish species have higher thermal tolerances (upper critical thermal maximum based on laboratory experiments) than “temperate” ones when acclimated at similar temperatures (41). This pattern does not contradict the fact that fish species in the tropics or at the warm range edges are most vulnerable to warming (42, 43). Ectotherms in warmer waters are still most sensitive to a rise in temperature not only because they have a narrower TSM (23, 25) but also because they are living closer to their upper thermal limits (17, 30). This study only shows that “tropical” fish species are relatively more thermally tolerant compared to their “temperate” counterparts when both are at the same acclimation temperatures. Our results may explain why this happens: Tropical species in shallow waters (<12.5 m) may be exposed to temperature variations that drive relatively larger ranges in biological rates in comparison with temperate species, which may ultimately increase selection of relatively high thermal tolerance in “tropical” fish species. Second, it was found that rocky and coral reef fishes generally fall into two thermal guilds, representing either warm (tropical) or cool (temperate) regions (44), such that fish species found exclusively at subtropical latitudes are rare. While sampling bias was initially implicated in this pattern (44), ongoing and systematic surveys have failed to reveal an exclusive subtropical shallow reef fish fauna, since this pattern was first noticed in 2017. Moreover, species richness trends from the equator polewards across the northern and southern hemisphere are bimodal and peak where tropical and temperate species overlap in occurrence (45–47). Our results thus suggest that an important direction for future research is to investigate the role of high temperature variability at short timescales in the subtropics as an alternative explanation for these macroecological patterns. Third, our findings can also explain why many marine teleosts do not conform to Rapoport's rule (48), which posits that species living at higher latitudes have a greater latitudinal range than those living at lower latitudes. An underlying assumption of Rapoport's rule is that more stable temperatures in the tropics translate to greater sensitivity to temperature variation, resulting in a latitudinal range that is narrower. However, this “rule” may not manifest in nature, especially across depths in the upper mixed layer of the ocean where most tropical reef species have been studied; indeed, we show that tropical systems can be as variable as temperate systems at short timescales.

Our work thus emphasizes the importance of considering the “ocean weather” in ecological research, which is missed by satellite SST data because of their coarse temporal resolutions. A number of studies from subtropical to tropical locations in Florida, the Caribbean, and eastern and central Pacific have noted that the interaction of thermal stratification in the water column with bottom topography leads to temperature variability at scales of minutes to hours that is equivalent in magnitude to variability across seasons (27–29, 32). Another study carried out near Moorea, in French Polynesia, using very high frequency (2 minutes) in situ temperature data reported that the temperature at different depths can vary greatly due to eddy-induced internal waves that can either increase or decrease the occurrence of marine heatwaves (49). The observed temperature variability in this system was a determining factor in whether the corals at shallow sites bleached or not. Indeed, eddies can have very different dynamics in different regions of the world (50), highlighting the importance of considering local oceanographic and geological factors which can buffer or propagate temperature variability. Besides the limitations posed by the coarse temporal resolution of satellite data for ecological research, another way in which SST data can miss the “ocean weather” conditions arises due to the fact that they only measure ocean temperatures at the surface, thereby overlooking ecologically important subsurface events (51).

Here, we find that short-term changes in “ocean weather” have great potential to impact organisms living in shallow depths across oceanic regions, including the tropics. Larger than expected temperature variation experienced by organisms at short timescales in tropical and subtropical oceans, and correspondingly high variation in temperature-dependent biological rates, may constrain organisms’ performance. Indeed, tropical and subtropical species may be even more sensitive to the changes in temperature in their respective regions compared to temperate species if short-term temperature variation has energetic and physiological consequences. Long-held assumptions about how patterns of environmental variability drive patterns in physiological sensitivity and vulnerability to future ocean climate change may require rethinking.

## Materials and methods

### Data collection

We began assembling the database in June 2020. Data were gathered from a variety of sources including: personal networking, broadcasted data requests on Twitter (now known as X), and from online data portals that provide public data access [e.g. ONC (https://www.oceannetworks.ca/), BODC (https://www.bodc.ac.uk/), and IMAS (https://data.imas.utas.edu.au/)]. The data were stored in a MySQL database, which allowed easier transfer of data to R 4.0.2 (52) for analysis through an R package called *RMySQL*, v0.10.23 (53). Sample time series are shown in Fig. 1, with information pertaining to these time series provided in Table 1.

**Table 1.** Information pertaining to the sample time series in Fig. 1.

Inset	Latitude (°)	Longitude (°)	Depth (m)	Logger used	Accuracy of sensor (°C)	Precision of sensor (°C)
a	−0.87	−82.58	8	HOBO v2 Water Temp Pro sensor, Onset	0.21	0.02
b	−12	55	1	Standard ATLAS SST sensor using YSI (Yellow Springs Instruments) thermistor 46006	0.03	0.001
c	−27.94	−48.55	12	HOBO Pendant Temperature Data Logger UA-002	0.53	0.14
d	−20.90	115.46	4.9	Sensus Ultra loggers (produced by ReefNet Inc., Canada)	0.8	0.01
e	−42.12	148.09	8.3	HOBO v2 Water Temp Pro sensor, Onset	0.21	0.02
f	−41.50	−72.31	1.5	Information on loggers could not be obtained	No info	No info

### Quality control

Data from instruments deployed on gliders or water column profilers were not included as we only aimed for fixed station deployments. To ensure that only subtidal samples were analyzed, we excluded time series that contained aerial exposure during low tides. This was determined through direct communication with the data providers, who flagged data that were exposed to air during low tides. Data that contained irregular frequencies, such as expected following equipment failure, were either corrected accordingly (filled with “NA” values) or discarded. Time series were subjected to a further quality control process including: removal of duplicated measurements and data anomalies assumed to be artifacts (such as unusually extreme temperatures).

### Filtering

We only used time series with measurement frequencies of 1 hour or less that spanned at least half a year in duration. The longest time series had a duration of 29 years. We also filtered the data according to depth, which ranged from the sea surface to depths shallower than 12.5 m. Initially, we aimed for depths shallower than 10 m, but because depth estimates can vary due to tidal height, we opted for depths shallower than 12.5 m to include data that are essentially at 10 m depth but are listed at slightly deeper depths due to the effect of tides. The resulting dataset comprised 492 time series, containing 68,110,162 temperature measurements, spread across 429 locations with unique coordinates (i.e. since some locations extended to several depths) and spanning between −53° and 55° of latitude (Fig. 1). The climate classifications of these 492 time series were as follows: 169 tropical, 179 subtropical, and 144 temperate.

### Climate classification

We assigned each time series into one of these three climate regions: “tropical” representing latitudes <20°, “subtropical” distinguishing as ≥20° and ≤35°, and “temperate” representing >35° and ≤55°. We recognized that latitude alone cannot be used to demarcate between the different climate classifications (54). However, since the goal of this study was to test the “tropical temperature stability paradigm,” we followed protocols of previous studies where “tropical” was classified according to a latitudinal threshold alone [e.g. (41, 55, 56)], and we selected a conservative threshold which was consistent with as many studies as possible. We further distinguished “subtropical” systems because the seasonal variability in the subtropics was expected to be less than that at higher latitudes, but greater than that at lower latitudes [e.g. (20)].

### Temperature range quantification

To test for differences in temperature variability between ocean regions, we first standardized our data to quarter-diurnal, semi-diurnal, diurnal, weekly, bi-weekly, monthly, and annual windows. These temporal windows represent different astronomical cycles (e.g. diurnal and lunar) that could affect or are known to affect the ocean's temperature. For instance, the lunar cycle affects ocean currents, which in turn can change the heat content at a particular point in the ocean (57). The selected temporal windows also span common biological rhythms (e.g. circadian and annual) of marine species (58, 59), allowing us to assess the effect of temperature variability on biological rates in these different time spans. For each temperature time series, we then calculated the total temperature range for each temporal window by subtracting the lowest value from the highest value recorded. For example, the diurnal range for a time series with a 30-minute sampling frequency would be calculated as the maximum minus the minimum temperature returned from 48 measurements. Thus, each time series returned multiple temperature range estimates. For instance, a time series that spanned 1 year in duration would contain 365 or 366 temperature range estimates for the diurnal temporal window. For all temporal windows, with the exception of the “Annual” one, we further subdivided time series that were longer than 1 year into 1-year subsamples, to standardize our time series that had vastly different durations (from half a year to 29 years). This step was not done for the “Annual” temporal window because the median and 90th percentile have to be calculated from at least three temperature range estimates, making it impossible to subdivide the time series in this case. For each of these subsamples, we then computed the median temperature range to estimate the central tendency and the 90th percentile to represent the extreme temperature range, repeating the same procedure for each temporal window. There were more locations being represented for the shorter temporal windows (quarter-diurnal to monthly) than there were for the “Annual” temporal window, because the durations of some time series were <3 years and these could therefore not be included for the calculation of the temperature range in the “Annual” window.

### Sensitivity tests

There were significant parts of some time series that contained missing values. To test whether the results were robust to these issues, we performed three sensitivity tests. First, we checked whether missing values could have been an issue (SI Appendix, Fig. S1), because calculation of the median and 90th percentile over a temporal window that has too little information can bias the results. We used a stricter threshold for the longer temporal windows (weekly to annual; see legend in SI Appendix, Fig. S1) since these contain less temperature range estimates per subsample (e.g. a 1-year subsample would contain 365 or 366 temperature range estimates for the diurnal temporal window and around 52 estimates for the weekly window), meaning that missing values have more potential to bias the median and 90th percentile because of less estimates. Second, we filtered out time series that were <3 years in duration (SI Appendix, Fig. S2). This implies that all the temporal windows, from quarter-diurnal to annual, had exactly the same samples of time series between them, because, for the “Annual” window, it was not possible to compute the median and 90th percentile for the time series that were <3 years in duration. We acknowledge that even three values might not be enough to compute the median and 90th percentile. However, in all three sensitivity tests, the trends for the annual windows were consistent; this is especially the case for the temperature range, where all the trends are similar and agree with the temperature stability paradigm in the tropics. The third sensitivity test combined the criteria of both the first and second tests (SI Appendix, Fig. S3). We also performed two additional sensitivity analyses to test whether our results are robust to: (i) different *E* values (see Materials and methods—Biological rate quantification for more details) and (ii) the removal of temperature data recorded in the open ocean (SI Appendix, Fig. S5). The latter sensitivity test was performed because the physical processes in the open ocean and on the continental shelf can be different (60). There were more in situ loggers in the open ocean in tropical regions compared to subtropical and temperate regions (Fig. 1), which could have biased our results. GEBCO bathymetry data (61) were used to obtain depth estimates of the ocean floor close to our loggers, and loggers that were located close to grid points where the ocean floor was deeper than 200 m were classified as “open ocean.”

### Biological rate quantification

We estimated biological rates on the rise component of the thermal performance curve based on the exponential relationship with temperature according to the equation:


$$\mathrm{Biological}\;\mathrm{rate}=R_{o}e^{-\frac{E}{kT}}$$

where *E* is the activation energy (eV), *k* is the Boltzmann's constant (8.617 × 10^−5^ eV *K*^−1^), *T* is the absolute temperature in kelvin, and *R*_0_ is an organism- and state-dependent scaling coefficient (62, 63).

We selected an *E* value of 0.63 eV that represents an average value for different organisms, from small aerobic microbes to larger animals like fishes and reptiles (24, 34). An *R*_0_ value of *e*^10.38^ was used as a typical value for fish (23). Because *E* can vary across species (23, 24), we performed another sensitivity analysis whereby we used an *E* value of 0.433 eV—a typical *E* value for fish (23)—to test the robustness of our results (see SI Appendix, Fig. S4). In practice, both *E* and *R*_0_ would be changed depending on the species (23). However, because the objective of this paper was to test the effect of temperature variability alone on biological rates, we kept *R*_0_ constant for simplicity. In other words, changing *R*_0_ would simply rescale the rest of the equation, making it useful only if we were interested in linking our estimates of biological rates to empirical data versus quantifying temperature-dependent scaling.

From the above equation, the difference in biological rates for each temporal window was then calculated as:


$$\mathrm{Difference}\;\mathrm{in}\;\mathrm{biological}\;\mathrm{rates}=R_{o}e^{-\frac{E}{kT_{\max}}}-R_{o}e^{-\frac{E}{kT_{\min}}}$$

where $T_{max}$ and $T_{min}$ are the maximum and minimum temperatures (in kelvin) over each temporal window, respectively.

### Statistical modeling

To test whether different ranges of temperature and biological rates occur between regions (tropical, subtropical, and temperate), we implemented a hierarchical modeling approach using Bayesian inference with Stan (64) and the “brms” package (65) within the R programming environment (52). For each index of variability (range of temperature and biological rates) derived from each temporal window (quarter-diurnal, semi-diurnal, diurnal, weekly, bi-weekly, monthly, and annual), we specified models by ascribing variation among the data to region (i.e. “climate_classification”) and instrument depth (i.e. “depth_in_m”). We also grouped variation among geographically proximate locations to account for spatial autocorrelation by including a random intercept (i.e. “spatial_blocks”) for sampling sites falling within a 174-km radius of each other (66) (see Supporting Information Text for more details). For all temporal windows with the exception of the “Annual” ones, we used an additional level (“plot_id”) of random effects in a nested design because the time series that were longer than 1 year in duration were subdivided into 1-year subsamples (see Materials and methods—Temperature range quantification). We specified a gamma error distribution with a log link to ensure normal residual distributions for the positively skewed, nonnegative data distributions (65, 67). All models were fit using 2,000 iterations across four chains with the first 1,000 iterations for each chain discarded as a warm-up and did not specify any priors, meaning that a flat prior was used by default (65). We checked convergence of models with visual inspections of trace plots, ensuring that $\hat{R}$ was <1.05 (implying model convergence) and that there was correspondence between observed and fitted values (65, 68) (see SI Appendix, Figs. S9, S11, S13, and S15). To estimate effect sizes of the climate classifications (tropical, subtropical, and temperate), we took the average from expected values of the posterior predictive distribution for each region classification while holding “depth” constant at its average values, using the “emmeans” package (69). To infer differences among regions, we contrasted these expected values between region pairs, taking the mean of these new distributions as the marginal effect size and considering the evidence as strong (indicated by the presence of asterisks in Figs. 2 to 5) if the 0.95 credible intervals did not include zero and weak if they did include zero (see SI Appendix, Figs. S10, S12, S14, and S16). The summary tables of the Bayesian models are presented in SI Appendix, Tables S1–S28.

### Generalized additive mixed-effects models

The trends across absolute latitude were visualized using GAMM using the “gamm4” package (70) within the R programming environment (52). For each index of variability (range of temperature and biological rates) derived from each temporal window (quarter-diurnal, semi-diurnal, diurnal, weekly, bi-weekly, monthly, and annual), we specified models by ascribing variation among the data to the absolute latitude (decimal degrees) and instrument depth (meters). We also grouped variation among geographically proximate locations to account for spatial autocorrelation by including a random intercept (i.e. “spatial_blocks”) for sampling sites falling within a 174-km radius of each other (66) (see Supporting Information Text for more details). For all temporal windows with the exception of the “Annual” ones, we used an additional level (“plot_id”) of random effects in a nested design because the time series that were longer than 1 year in duration were subdivided into 1-year subsamples (see Materials and methods—Temperature range quantification).

## Supplementary Material

pgae260_Supplementary_Data

## Data Availability

We provide the files containing the computed temperature ranges and difference in biological rates from the raw data, which were used to generate the figures (https://doi.org/10.6084/m9.figshare.21386430.v5). We used data from public data portals and private repositories. The metadata file in the above link lists which data are public and private. The websites where the public data were downloaded are also provided. The metadata file also contains other important information about the data, such as the coordinates at which the measurements were taken, the frequency of the data, and the time span of each record. The codes used to generate the results are available on GitHub (https://github.com/jmschuster/Ocean_temperature_variability_2022).
