# Supplementary material for: Ocean weather, biological rates, and unexplained global ecological patterns
Source: PNAS Nexus. 2024 Aug 6;3(8):pgae260. doi: 10.1093/pnasnexus/pgae260 (PMC11302846; doi:10.1093/pnasnexus/pgae260)
Supplement: pgae260_Supplementary_Data [file pgae260_supplementary_data.docx]

**Supporting Information for**

Ocean weather, biological rates and unexplained global ecological patterns

Darren L.C.Y. Li Shing Hiung^*^, Jasmin M. Schuster, Murray I. Duncan, Nicholas L. Payne, Brian Helmuth, Jackson W.F. Chu, Julia K. Baum, Viviana Brambilla, John Bruno, Sarah W. Davies, Maria Dornelas, Patrick Gagnon, Tamar Guy-Haim, Jennifer M. Jackson, James J. Leichter, Joshua S. Madin, Zachary L. Monteith, Ana M. Queirós, Eric V.C. Schneider, Samuel Starko, Brendan S. Talwar, Alex S.J. Wyatt, Hannah E. Aichelman, Nathaniel Bensoussan, Carlo Caruso, Karl Castillo, Francis Choi, Yun-Wei Dong, Joaquim Garrabou, Dorian Guillemain, Nicholas Higgs, Yuwu Jiang, Diego K. Kersting, David J. Kushner, Guilherme O. Longo, Christopher Neufeld, Marion Peirache, Tim Smyth, Joshua L. Sprague, Gaëlle Urvoy, Frederic Zuberer, and Amanda E. Bates

*Darren L.C.Y. Li Shing Hiung

Email: [chongyoune@gmail.com](mailto:chongyoune@gmail.com)

**This PDF file includes:**

Supporting text

Figures S1 to S16

Tables S1 to S30

SI References

Additional supporting references (for data and *R* packages)

Supporting Information Text

**Spatial decorrelation scales of ocean temperature.** In our models, we accounted for spatial autocorrelation of ocean temperature by adding a random intercept for time series that are within a 174 km radius of each other. This number was estimated from Hosoda & Kawamura (2004) (Ref. [(1)](https://www.zotero.org/google-docs/?BlZfhw)), whose results show that for SST the zonal scale is between 1.5-3.0 degrees and the meridional scale is between 1.2-2.0 degrees. Each degree of latitude is roughly constant at around 111 km, while the degree of longitude varies based on the location on the Earth; at the equator, it is around 111 km and it decreases to 0 km towards the poles. Since our data spanned between -53 and 55 degrees of latitude (Fig. 1), we can calculate the minimum distance in km between 1 degree of longitude at an average of ± 54 degrees of latitude (65 km; [Latitude/Longitude Distance Calculator (noaa.gov)](https://www.nhc.noaa.gov/gccalc.shtml)). Thus, we take an average value for the distance between 1 degree of longitude [(111 km + 65 km) / 2], because our data was evenly spread along the latitudinal range (Fig. 1). Taking the average of 111 km (for 1 degree of latitude) and 88 km (for 1 degree of longitude on average) gives 99.5 km. The overlap of zonal and meridional scale is 1.5-2.0 degrees, and taking this average gives 1.75 degrees. Therefore, 1.75 degrees * 99.5 km results in 174 km.


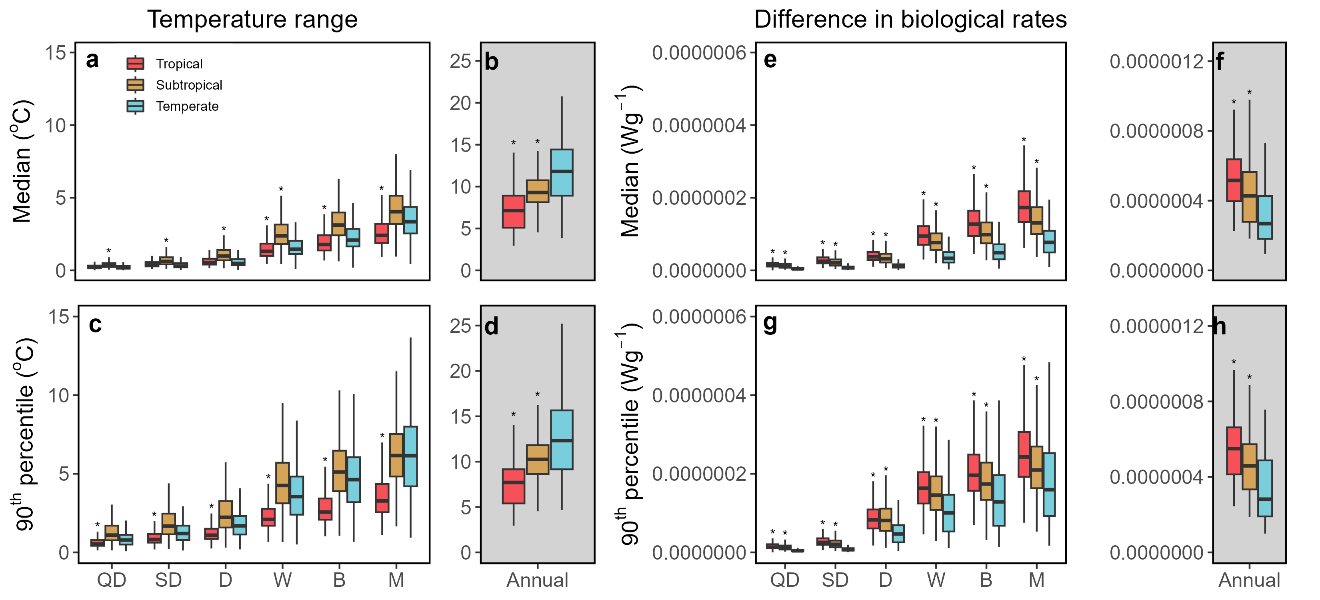


**Fig. S1** | **Results of the first sensitivity test showing box plots of the median and the 90^th^ percentile of the temperature range (a-d) and of the range of biological rates (e-h).** The different climate classifications are distinguished by colour: blue (temperate), yellow (subtropical) and red (tropical). The 7 temporal windows are shown on the x-axis: quarter-diurnal (QD), semi-diurnal (SD), diurnal (D), weekly (W), bi-weekly (B), monthly (M), and annual. Asterisks indicate that the Bayesian models showed strong evidence (i.e., the 0.95 credible intervals do not include zero) that tropical and/or subtropical regions differed from temperate regions. The results shown here used a subset of the data in Figs. 2 to 5 of the main paper, as a sensitivity test to check the robustness of our results. For each temporal window, the median and 90^th^ percentile were calculated only if the temperature records did not contain more than the following percentage of missing values over the period of the temporal window: i) Quarter-diurnal - 10%, ii) Semi-diurnal - 10%, iii) Diurnal - 10%, iv) Weekly - 30%, v) Bi-weekly - 30%, vi) Monthly - 30%, and vii) Annual - 30%. In addition, for weekly, bi-weekly, monthly and annual, there had to be more than 5, 11, 27 and 330 days in the temporal window respectively.


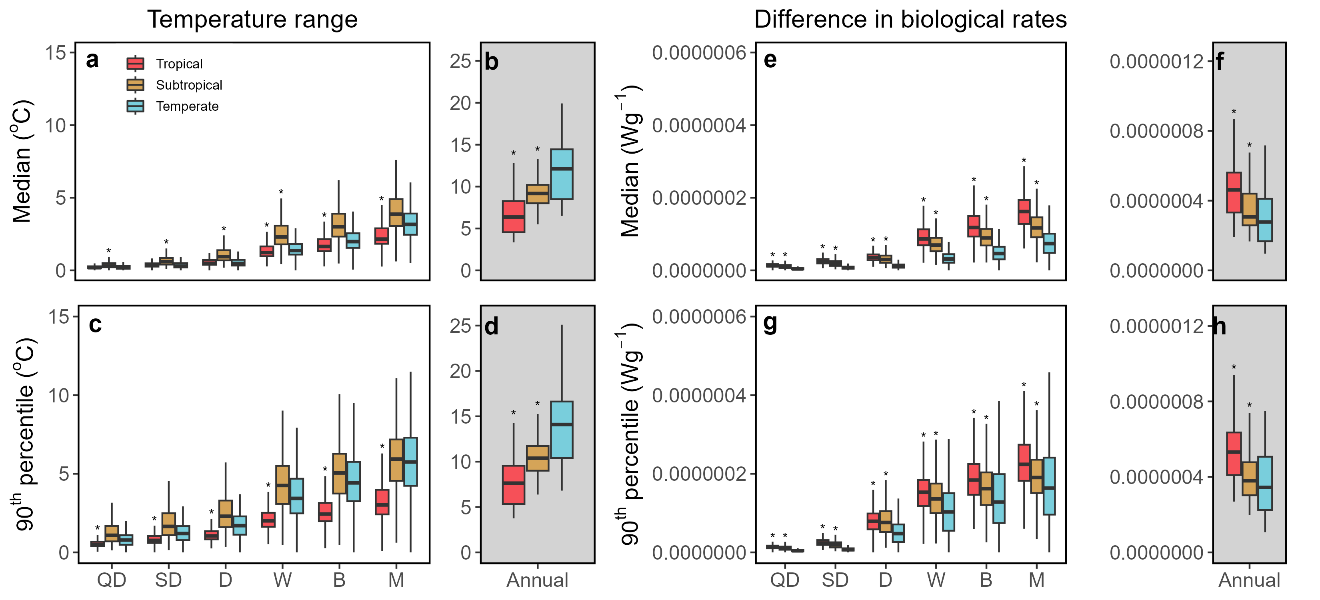


**Fig. S2 | Results of the second sensitivity test showing box plots of the median and the 90^th^ percentile of the temperature range (a-d) and of the range of biological rates (e-h).** The different climate classifications are distinguished by colour: blue (temperate), yellow (subtropical) and red (tropical). The 7 temporal windows are shown on the x-axis: quarter-diurnal (QD), semi-diurnal (SD), diurnal (D), weekly (W), bi-weekly (B), monthly (M), and annual. Asterisks indicate that the Bayesian models showed strong evidence (i.e., the 0.95 credible intervals do not include zero) that tropical and/or subtropical regions differed from temperate regions. The results shown here used a subset of the data in Figs. 2 to 5 of the main paper, as a sensitivity test to check the robustness of our results. Time series that were less than 3 years in duration were removed, such that all temporal windows had the same locations being represented.


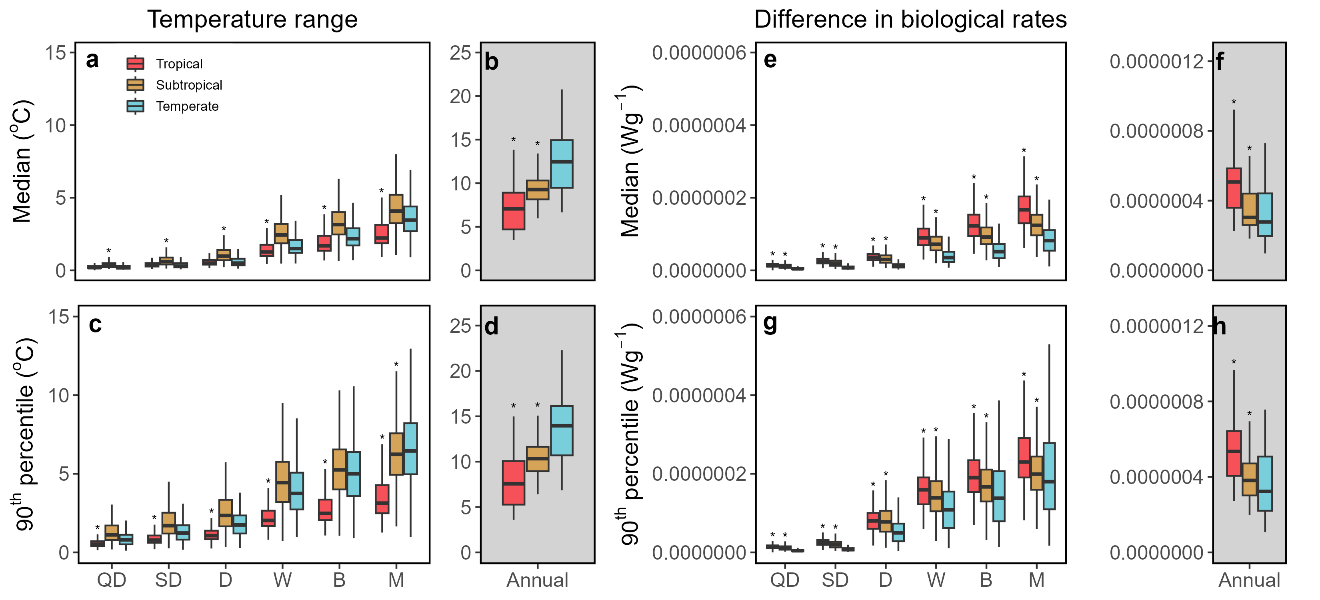


**Fig. S3 |** **Results of the third sensitivity test showing box plots of the median and the 90^th^ percentile of the temperature range (a-d) and of the range of biological rates (e-h).** The different climate classifications are distinguished by colour: blue (temperate), yellow (subtropical) and red (tropical). The 7 temporal windows are shown on the x-axis: quarter-diurnal (QD), semi-diurnal (SD), diurnal (D), weekly (W), bi-weekly (B), monthly (M), and annual. Asterisks indicate that the Bayesian models showed strong evidence (i.e., the 0.95 credible intervals do not include zero) that tropical and/or subtropical regions differed from temperate regions. The results shown here used a subset of the data in Figs. 2 to 5 of the main paper, as a sensitivity test to check the robustness of our results. These results met the criteria of the results in both Figs. S1 and S2.


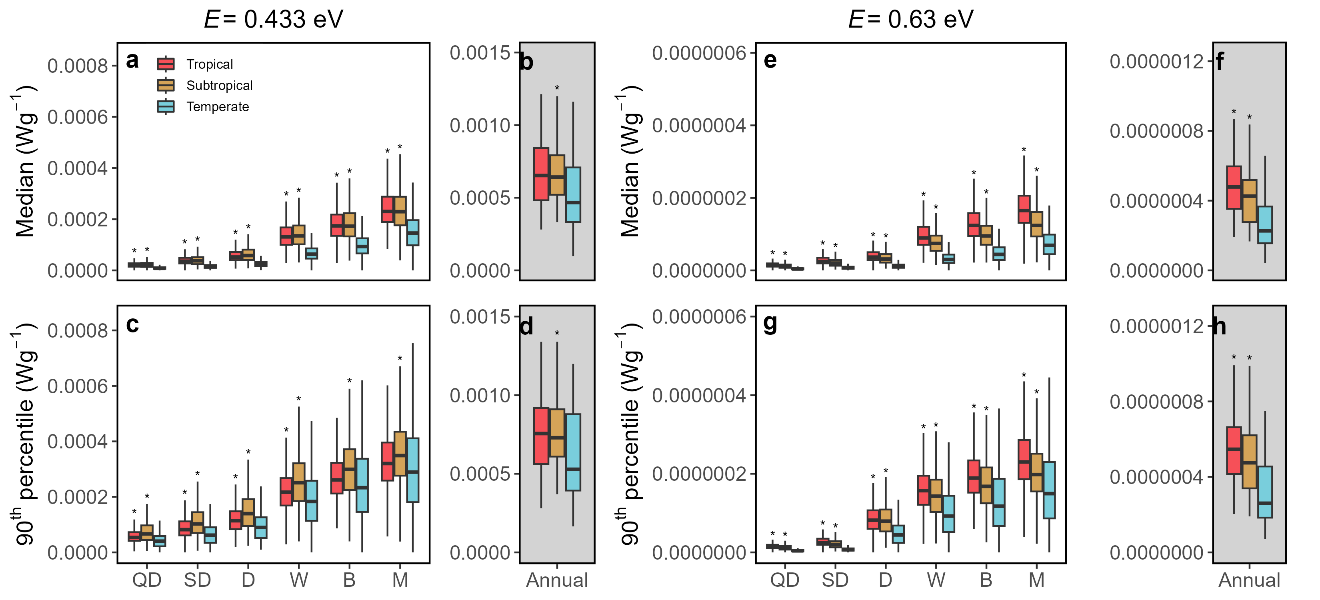


**Fig. S4 |** **Results of the fourth sensitivity test showing box plots of the median and the 90^th^ percentile of the difference in biological rates using an *E* value of 0.433 eV (a-d) and an *E* value of 0.63 (e-h).** The different climate classifications are distinguished by colour: blue (temperate), yellow (subtropical) and red (tropical). The 7 temporal windows are shown on the x-axis: quarter-diurnal (QD), semi-diurnal (SD), diurnal (D), weekly (W), bi-weekly (B), monthly (M), and annual. Asterisks indicate that the Bayesian models showed strong evidence (i.e., the 0.95 credible intervals do not include zero) that tropical and/or subtropical regions differed from temperate regions. The results shown here used the same data as in Figs. 4 and 5 of the main paper, but with different *E* values, as a sensitivity test to check the robustness of our results.


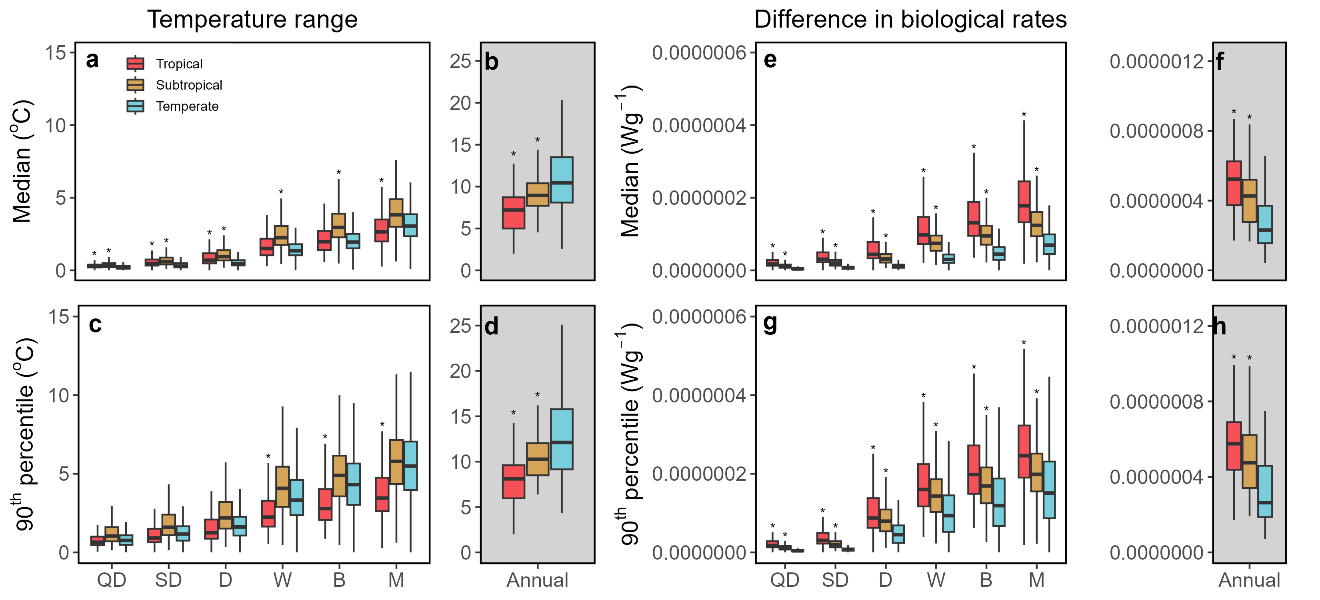


**Fig. S5** | **Results of the fifth sensitivity test showing box plots of the median and the 90^th^ percentile of the temperature range (a-d) and of the range of biological rates (e-h).** The different climate classifications are distinguished by colour: blue (temperate), yellow (subtropical) and red (tropical). The 7 temporal windows are shown on the x-axis: quarter-diurnal (QD), semi-diurnal (SD), diurnal (D), weekly (W), bi-weekly (B), monthly (M), and annual. Asterisks indicate that the Bayesian models showed strong evidence (i.e., the 0.95 credible intervals do not include zero) that tropical and/or subtropical regions differed from temperate regions. The results shown here used a subset of the data in Figs. 2 to 5 of the main paper, as a sensitivity test to check the robustness of our results. Data from loggers that were deployed in the open ocean were removed to check whether our results in the tropical oceans could have been biased because of the large number of loggers in the open ocean there.


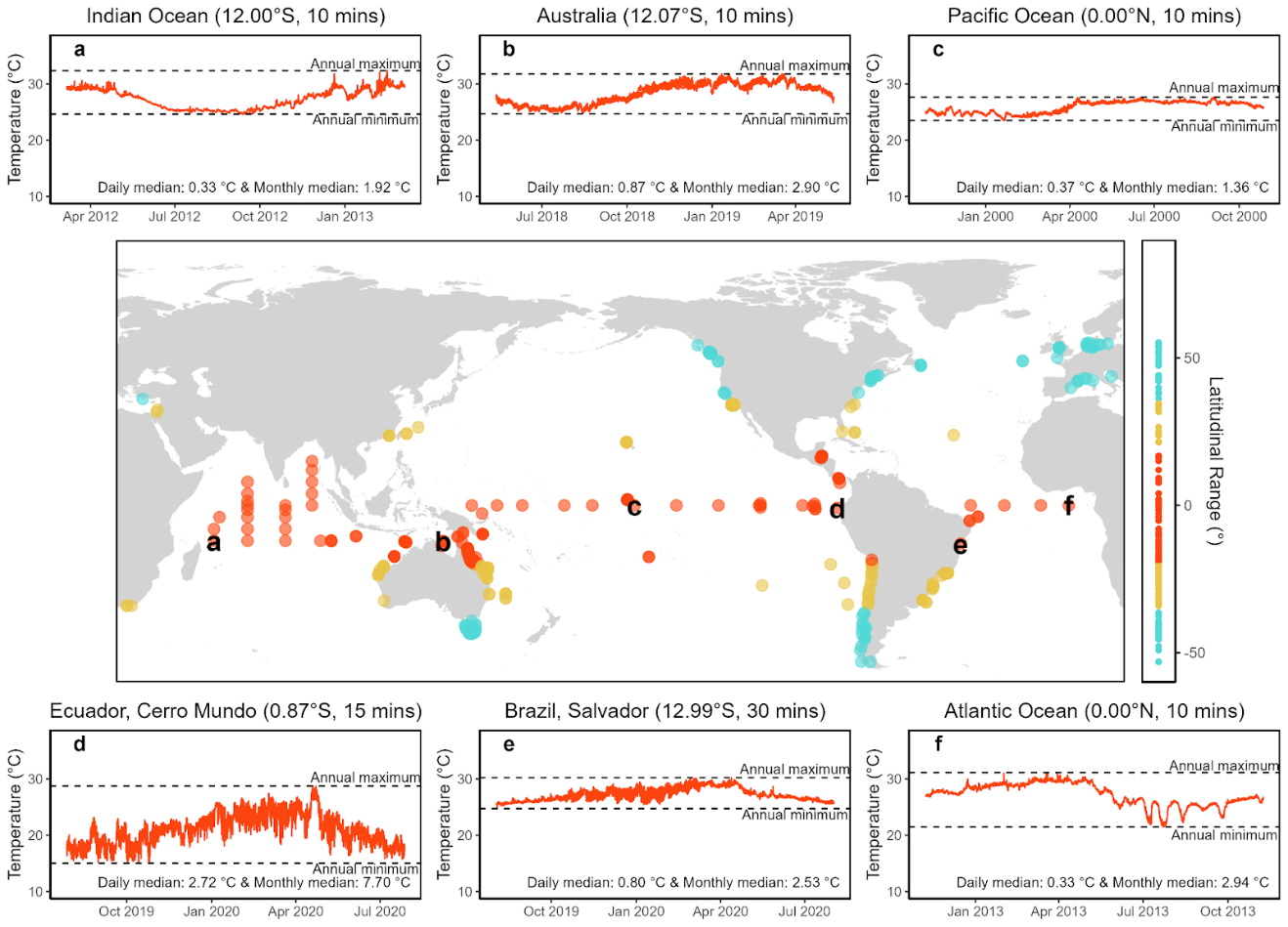


**Fig. S6 |** **Additional examples of time series from tropical regions.** This figure is similar to Fig. 1 of the main paper, with additional examples of time series shown from tropical regions. Insets (**a**-**f**) correspond to the temperature time series at the locations shown by the respective letters. These sample time series help to visualize the range of temporal variability of tropical locations at the shorter temporal windows. The numbers between brackets in the title of each inset indicate the latitude and the measurement frequency of the respective time series. The median temperature ranges over the diurnal and monthly temporal windows are also shown for each inset. The y-axes of all 6 insets have the same range, for comparability.


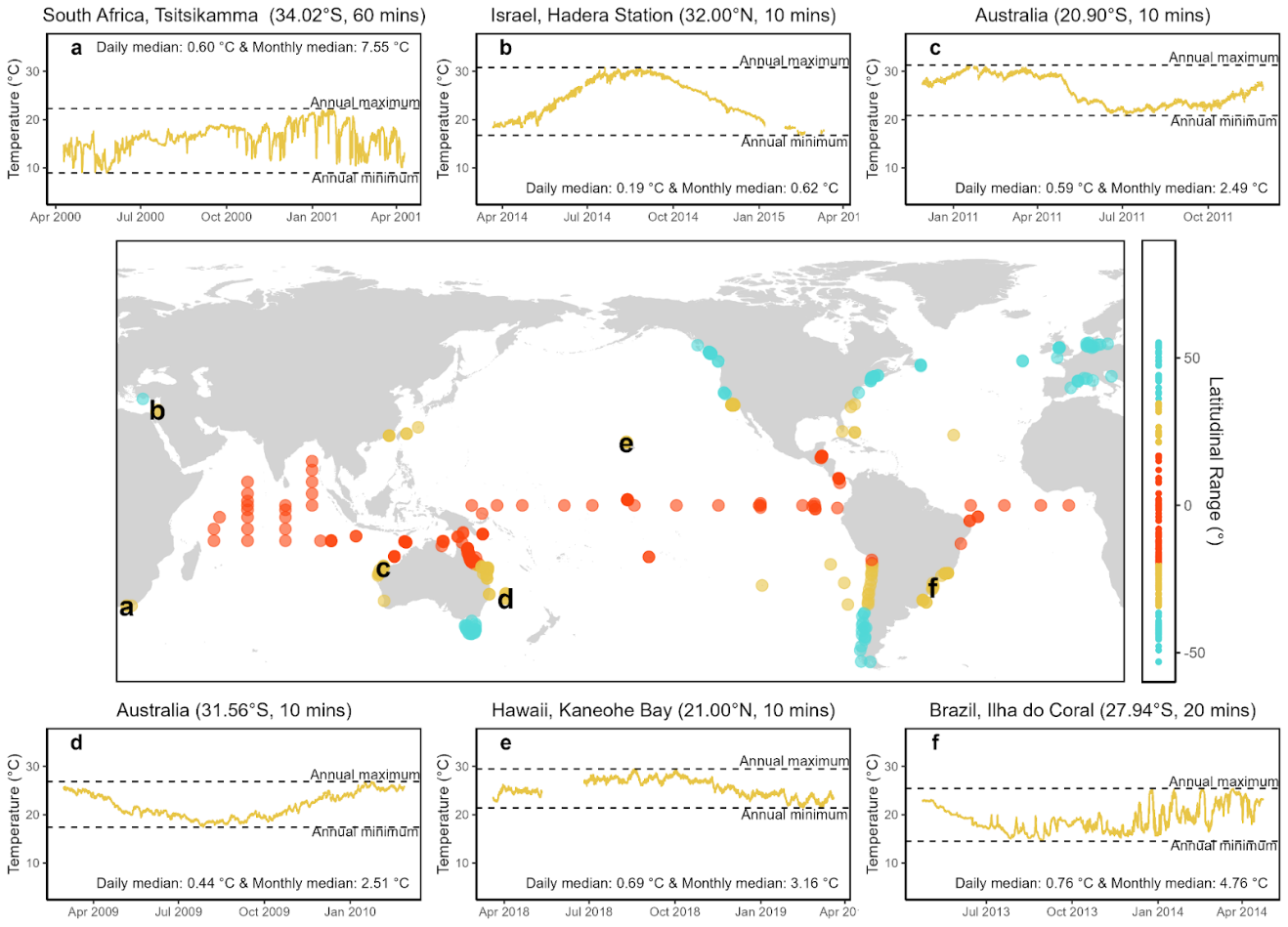


**Fig. S7 |** **Additional examples of time series from subtropical regions.** This figure is similar to Fig. 1 of the main paper, with additional examples of time series shown from subtropical regions. Insets (**a**-**f**) correspond to the temperature time series at the locations shown by the respective letters. These sample time series help to visualize the range of temporal variability of subtropical locations at the shorter temporal windows. The numbers between brackets in the title of each inset indicate the latitude and the measurement frequency of the respective time series. The median temperature ranges over the diurnal and monthly temporal windows are also shown for each inset. The y-axes of all 6 insets have the same range, for comparability.


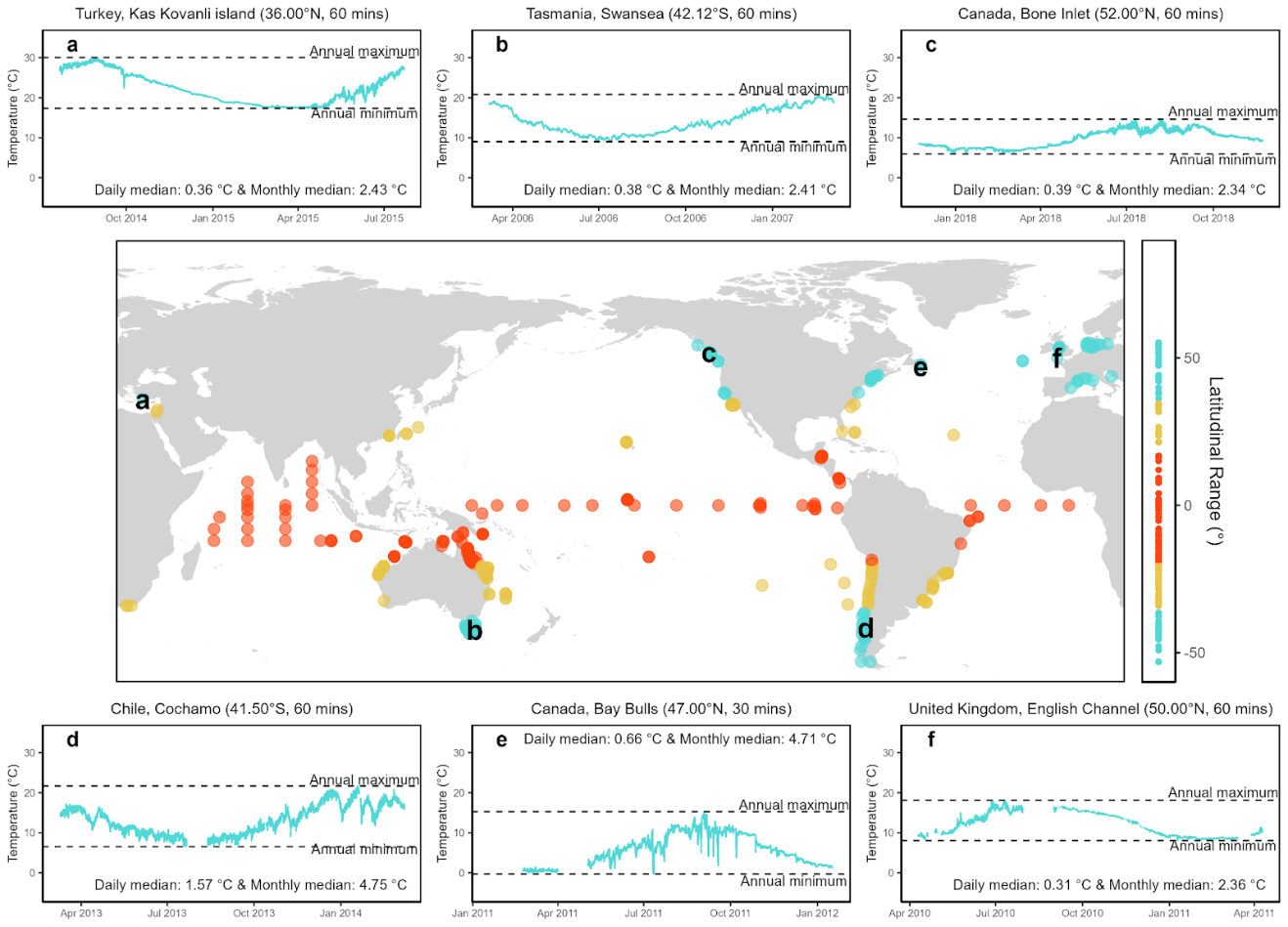


**Fig. S8 |** **Additional examples of time series from temperate regions.** This figure is similar to Fig. 1 of the main paper, with additional examples of time series shown from temperate regions. Insets (**a**-**f**) correspond to the temperature time series at the locations shown by the respective letters. These sample time series help to visualize the range of temporal variability of temperate locations at the shorter temporal windows. The numbers between brackets in the title of each inset indicate the latitude and the measurement frequency of the respective time series. The median temperature ranges over the diurnal and monthly temporal windows are also shown for each inset. The y-axes of all 6 insets have the same range, for comparability.


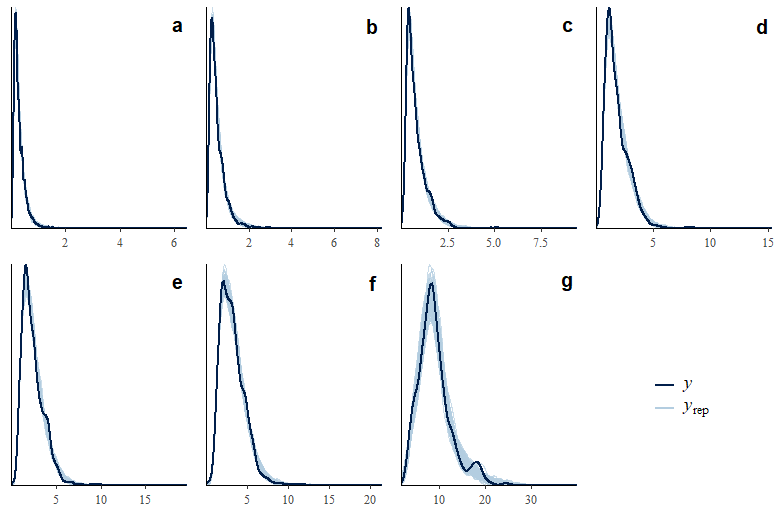


**Fig. S9 | Posterior predictive checks for the median of the temperature range, visually checked to ensure there was correspondence between the observed and fitted values.** Panels **a**-**g** represent the plots for the different temporal windows: quarter-diurnal (**a**), semi-diurnal (**b**), diurnal (**c**), weekly (**d**), bi-weekly (**e**), monthly (**f**), and annual (**g**). y represents the distribution of the observed value (dark blue), and y_rep_ represents the distributions of 100 of posterior draws from the model (light blue).

**
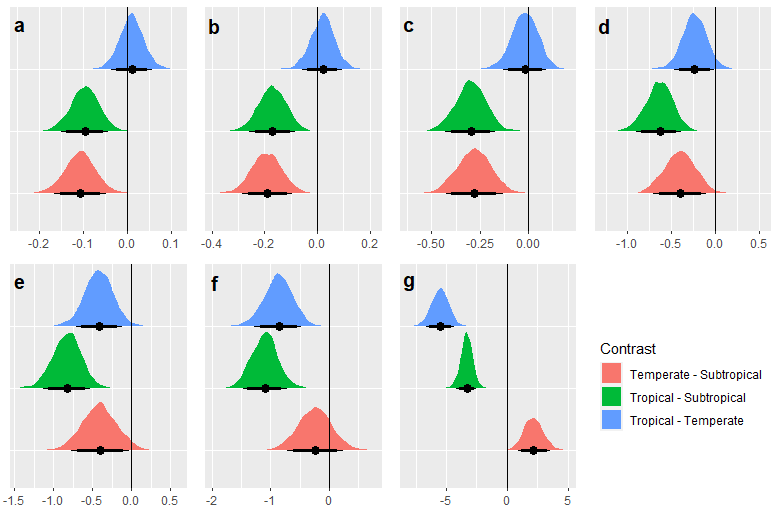
**

**Fig. S10 | Distributions of the differences of the marginal effect sizes of the median of the temperature range between region pairs.** The marginal effect sizes of each climate classification was obtained from the average of the expected values of the posterior predictive distribution of the Bayesian models, holding “depth” constant at its average values, using the “emmeans” package [(2)](https://www.zotero.org/google-docs/?50HDiI). The difference of the marginal effect sizes for each region pair is colour-coded: temperate and subtropical (red), tropical and subtropical (green), and tropical and temperate (blue). Panels **a**-**g** represent these distributions for the different temporal windows: quarter-diurnal (**a**), semi-diurnal (**b**), diurnal (**c**), weekly (**d**), bi-weekly (**e**), monthly (**f**), and annual (**g**). There is strong evidence that the marginal effect sizes of the region pairs are different from one another if the 0.95 credible interval does not include zero.

**
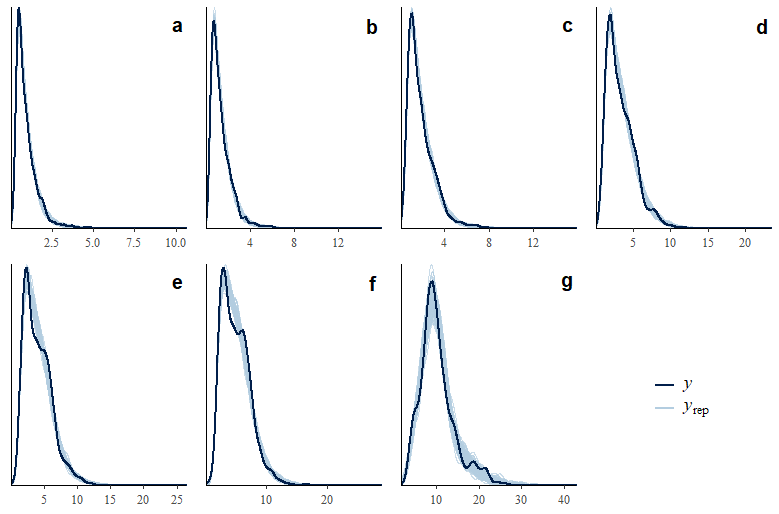
**

**Fig. S11 | Posterior predictive checks for the 90^th^ percentile of the temperature range, visually checked to ensure there was correspondence between the observed and fitted values.** Panels **a**-**g** represent the plots for the different temporal windows: quarter-diurnal (**a**), semi-diurnal (**b**), diurnal (**c**), weekly (**d**), bi-weekly (**e**), monthly (**f**), and annual (**g**). y represents the distribution of the observed value (dark blue), and y_rep_ represents the distributions of 100 of posterior draws from the model (light blue).

**
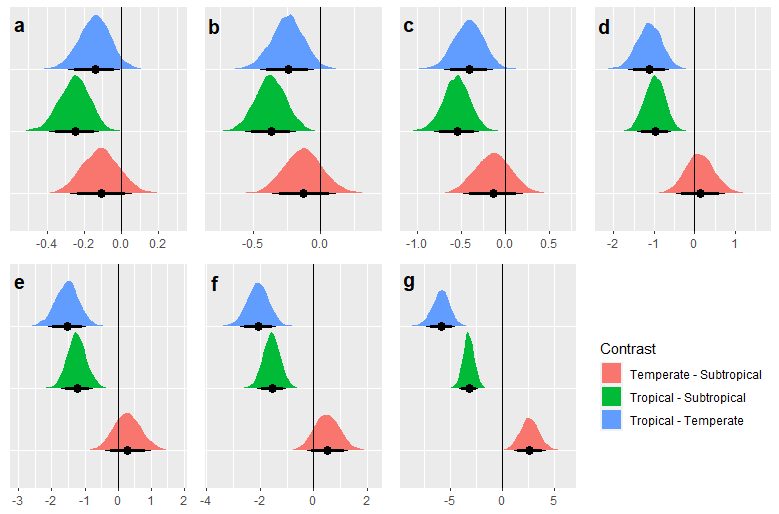
**

**Fig. S12 | Distributions of the differences of the marginal effect sizes of the 90^th^ percentile of the temperature range between region pairs.** The marginal effect sizes of each climate classification was obtained from the average of the expected values of the posterior predictive distribution of the Bayesian models, holding “depth” constant at its average values, using the “emmeans” package [(2)](https://www.zotero.org/google-docs/?DhYWYd). The difference of the marginal effect sizes for each region pair is colour-coded: temperate and subtropical (red), tropical and subtropical (green), and tropical and temperate (blue). Panels **a**-**g** represent these distributions for the different temporal windows: quarter-diurnal (**a**), semi-diurnal (**b**), diurnal (**c**), weekly (**d**), bi-weekly (**e**), monthly (**f**), and annual (**g**). There is strong evidence that the marginal effect sizes of the region pairs are different from one another if the 0.95 credible interval does not include zero.

**
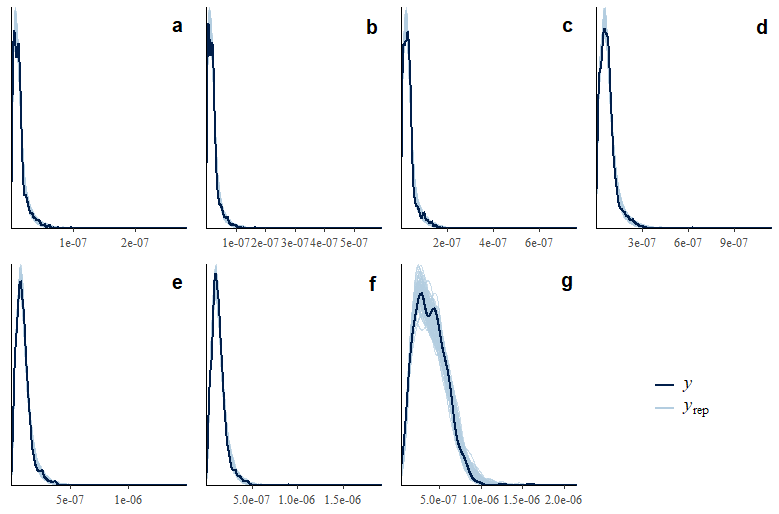
**

**Fig. S13 | Posterior predictive checks for the median of the difference in biological rates, visually checked to ensure there was correspondence between the observed and fitted values.** Panels **a**-**g** represent the plots for the different temporal windows: quarter-diurnal (**a**), semi-diurnal (**b**), diurnal (**c**), weekly (**d**), bi-weekly (**e**), monthly (**f**), and annual (**g**). y represents the distribution of the observed value (dark blue), and y_rep_ represents the distributions of 100 of posterior draws from the model (light blue).

**
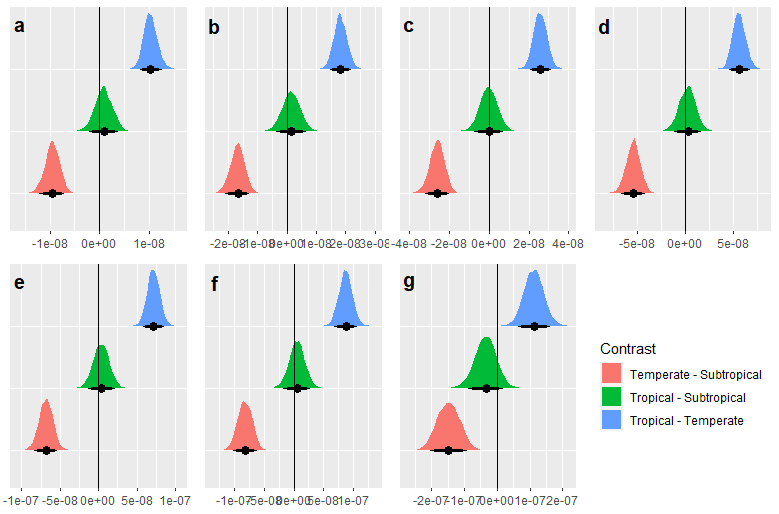
**

**Fig. S14 | Distributions of the differences of the marginal effect sizes of the median of the difference in biological rates between region pairs.** The marginal effect sizes of each climate classification was obtained from the average of the expected values of the posterior predictive distribution of the Bayesian models, holding “depth” constant at its average values, using the “emmeans” package [(2)](https://www.zotero.org/google-docs/?hChxQA). The difference of the marginal effect sizes for each region pair is colour-coded: temperate and subtropical (red), tropical and subtropical (green), and tropical and temperate (blue). Panels **a**-**g** represent these distributions for the different temporal windows: quarter-diurnal (**a**), semi-diurnal (**b**), diurnal (**c**), weekly (**d**), bi-weekly (**e**), monthly (**f**), and annual (**g**). There is strong evidence that the marginal effect sizes of the region pairs are different from one another if the 0.95 credible interval does not include zero.

**
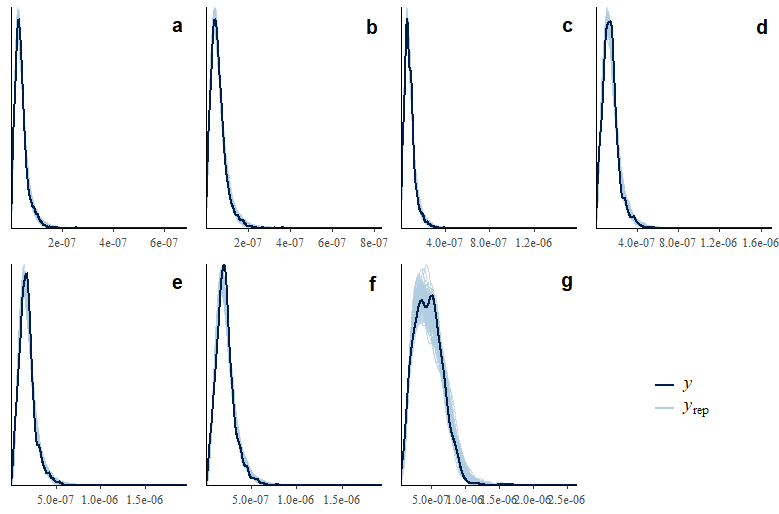
**

**Fig. S15 | Posterior predictive checks for the 90^th^ percentile of the difference in biological rates, visually checked to ensure there was correspondence between the observed and fitted values.** Panels **a**-**g** represent the plots for the different temporal windows: quarter-diurnal (**a**), semi-diurnal (**b**), diurnal (**c**), weekly (**d**), bi-weekly (**e**), monthly (**f**), and annual (**g**). y represents the distribution of the observed value (dark blue), and y_rep_ represents the distributions of 100 of posterior draws from the model (light blue).

**
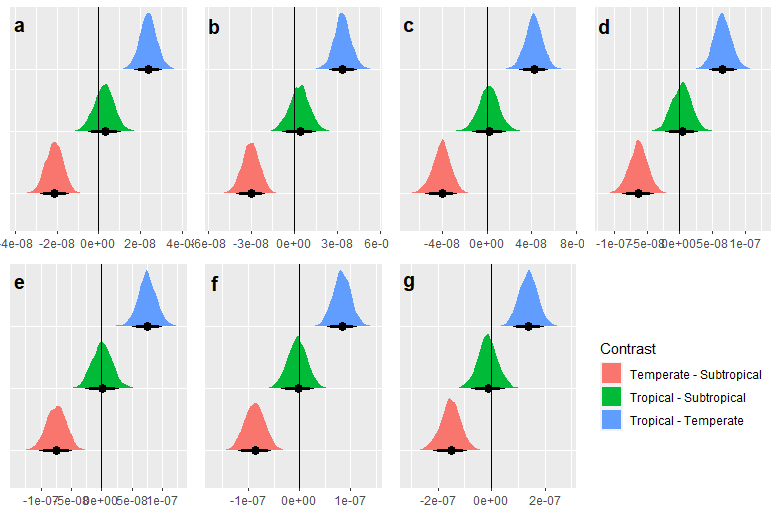
**

**Fig. S16 | Distributions of the differences of the marginal effect sizes of the 90^th^ percentile of the difference in biological rates between region pairs.** The marginal effect sizes of each climate classification was obtained from the average of the expected values of the posterior predictive distribution of the Bayesian models, holding “depth” constant at its average values, using the “emmeans” package [(2)](https://www.zotero.org/google-docs/?16uFMM). The difference of the marginal effect sizes for each region pair is colour-coded: temperate and subtropical (red), tropical and subtropical (green), and tropical and temperate (blue). Panels **a**-**g** represent these distributions for the different temporal windows: quarter-diurnal (**a**), semi-diurnal (**b**), diurnal (**c**), weekly (**d**), bi-weekly (**e**), monthly (**f**), and annual (**g**). There is strong evidence that the marginal effect sizes of the region pairs are different from one another if the 0.95 credible interval does not include zero.

**Table S1 | Summary tables of the Bayesian model for the quarter-diurnal median of temperature range.** To test whether the quarter-diurnal medians of temperature range vary significantly between regions (tropical, subtropical and temperate), we implemented a hierarchical modelling approach using Bayesian inference with Stan [(3)](https://www.zotero.org/google-docs/?w8Gld1) and the “brms” package [(4)](https://www.zotero.org/google-docs/?gPBc1y) within the R programming environment [(5)](https://www.zotero.org/google-docs/?pbHcLd). We specified models by ascribing variation among the data to “climate_classification” and “depth_in_m”, and grouped variation among geographically proximate locations to account for spatial autocorrelation by including a random intercept (“spatial_blocks”) for sampling sites falling within a 174 km radius of each other [(1)](https://www.zotero.org/google-docs/?UoAPbl). We used an additional level (“plot_id”) of random effects in a crossed design because the time series that were longer than 1 year in duration were divided into 1-year subsamples. Draws were sampled using sampling (NUTS). For each parameter, Bulk_ESS and Tail_ESS are effective sample size measures, and Rhat is the potential scale reduction factor on split chains (at convergence, Rhat = 1).

**Group-Level Effects**

| ~spatial_blocks (Number of levels: 120) | | | | | | | |
| --- | --- | --- | --- | --- | --- | --- | --- |
|  | Estimate | Est.Error | l-95% CI | u-95% CI | Rhat | Bulk_ESS | Tail_ESS |
| sd(Intercept) | 0.46 | 0.05 | 0.36 | 0.57 | 1.00 | 937 | 1192 |
| ~spatial_blocks:plot_id (Number of levels: 486) | | | | | | | |
|  | Estimate | Est.Error | l-95% CI | u-95% CI | Rhat | Bulk_ESS | Tail_ESS |
| sd(Intercept) | 0.43 | 0.02 | 0.39 | 0.46 | 1.00 | 761 | 1362 |

**Population-Level Effects**

|  | Estimate | Est.Error | l-95% CI | u-95% CI | Rhat | Bulk_ESS | Tail_ESS |
| --- | --- | --- | --- | --- | --- | --- | --- |
| Intercept | -0.98 | 0.10 | -1.17 | -0.80 | 1.00 | 961 | 1698 |
| climate_classificationTemperate | -0.41 | 0.14 | -0.67 | -0.13 | 1.00 | 1113 | 1726 |
| climate_classificationTropical | -0.37 | 0.11 | -0.59 | -0.15 | 1.00 | 1162 | 1949 |
| depth_in_m | -0.05 | 0.01 | -0.06 | -0.03 | 1.00 | 839 | 1492 |

**Family Specific Parameters**

|  | Estimate | Est.Error | l-95% CI | u-95% CI | Rhat | Bulk_ESS | Tail_ESS |
| --- | --- | --- | --- | --- | --- | --- | --- |
| shape | 13.00 | 0.34 | 12.35 | 13.69 | 1.00 | 5843 | 2821 |

**Table S2 | Summary tables of the Bayesian model for the semi-diurnal median of temperature range.** To test whether the semi-diurnal medians of temperature range vary significantly between regions (tropical, subtropical and temperate), we implemented a hierarchical modelling approach using Bayesian inference with Stan [(3)](https://www.zotero.org/google-docs/?k6Rqzs) and the “brms” package [(4)](https://www.zotero.org/google-docs/?v6PSui) within the R programming environment [(5)](https://www.zotero.org/google-docs/?xv5X5N). We specified models by ascribing variation among the data to “climate_classification” and “depth_in_m”, and grouped variation among geographically proximate locations to account for spatial autocorrelation by including a random intercept (“spatial_blocks”) for sampling sites falling within a 174 km radius of each other [(1)](https://www.zotero.org/google-docs/?k0AfIA). We used an additional level (“plot_id”) of random effects in a crossed design because the time series that were longer than 1 year in duration were divided into 1-year subsamples. Draws were sampled using sampling (NUTS). For each parameter, Bulk_ESS and Tail_ESS are effective sample size measures, and Rhat is the potential scale reduction factor on split chains (at convergence, Rhat = 1).

**Group-Level Effects**

| ~spatial_blocks (Number of levels: 120) | | | | | | | |
| --- | --- | --- | --- | --- | --- | --- | --- |
|  | Estimate | Est.Error | l-95% CI | u-95% CI | Rhat | Bulk_ESS | Tail_ESS |
| sd(Intercept) | 0.42 | 0.05 | 0.32 | 0.52 | 1.01 | 646 | 1139 |
| ~spatial_blocks:plot_id (Number of levels: 487) | | | | | | | |
|  | Estimate | Est.Error | l-95% CI | u-95% CI | Rhat | Bulk_ESS | Tail_ESS |
| sd(Intercept) | 0.43 | 0.02 | 0.40 | 0.47 | 1.01 | 852 | 1853 |

**Population-Level Effects**

|  | Estimate | Est.Error | l-95% CI | u-95% CI | Rhat | Bulk_ESS | Tail_ESS |
| --- | --- | --- | --- | --- | --- | --- | --- |
| Intercept | -0.39 | 0.09 | -0.57 | -0.21 | 1.00 | 918 | 1346 |
| climate_classificationTemperate | -0.43 | 0.13 | -0.68 | -0.17 | 1.00 | 980 | 1532 |
| climate_classificationTropical | -0.37 | 0.11 | -0.58 | -0.16 | 1.01 | 1015 | 1458 |
| depth_in_m | -0.05 | 0.01 | -0.07 | -0.03 | 1.00 | 798 | 1320 |

**Family Specific Parameters**

|  | Estimate | Est.Error | l-95% CI | u-95% CI | Rhat | Bulk_ESS | Tail_ESS |
| --- | --- | --- | --- | --- | --- | --- | --- |
| shape | 12.99 | 0.35 | 12.31 | 13.66 | 1.00 | 5302 | 3347 |

**Table S3 | Summary tables of the Bayesian model for the diurnal median of temperature range.** To test whether the diurnal medians of temperature range vary significantly between regions (tropical, subtropical and temperate), we implemented a hierarchical modelling approach using Bayesian inference with Stan [(3)](https://www.zotero.org/google-docs/?2P4Hei) and the “brms” package [(4)](https://www.zotero.org/google-docs/?hSbQ8Q) within the R programming environment [(5)](https://www.zotero.org/google-docs/?uroI5g). We specified models by ascribing variation among the data to “climate_classification” and “depth_in_m”, and grouped variation among geographically proximate locations to account for spatial autocorrelation by including a random intercept (“spatial_blocks”) for sampling sites falling within a 174 km radius of each other [(1)](https://www.zotero.org/google-docs/?n8cVwi). We used an additional level (“plot_id”) of random effects in a crossed design because the time series that were longer than 1 year in duration were divided into 1-year subsamples. Draws were sampled using sampling (NUTS). For each parameter, Bulk_ESS and Tail_ESS are effective sample size measures, and Rhat is the potential scale reduction factor on split chains (at convergence, Rhat = 1).

**Group-Level Effects**

| ~spatial_blocks (Number of levels: 120) | | | | | | | |
| --- | --- | --- | --- | --- | --- | --- | --- |
|  | Estimate | Est.Error | l-95% CI | u-95% CI | Rhat | Bulk_ESS | Tail_ESS |
| sd(Intercept) | 0.41 | 0.05 | 0.32 | 0.52 | 1.00 | 894 | 1767 |
| ~spatial_blocks:plot_id (Number of levels: 487) | | | | | | | |
|  | Estimate | Est.Error | l-95% CI | u-95% CI | Rhat | Bulk_ESS | Tail_ESS |
| sd(Intercept) | 0.41 | 0.02 | 0.38 | 0.45 | 1.00 | 876 | 1742 |

**Population-Level Effects**

|  | Estimate | Est.Error | l-95% CI | u-95% CI | Rhat | Bulk_ESS | Tail_ESS |
| --- | --- | --- | --- | --- | --- | --- | --- |
| Intercept | 0.06 | 0.09 | -0.12 | 0.24 | 1.00 | 1170 | 1823 |
| climate_classificationTemperate | -0.41 | 0.14 | -0.67 | -0.13 | 1.00 | 1265 | 1691 |
| climate_classificationTropical | -0.43 | 0.11 | -0.64 | -0.21 | 1.00 | 1369 | 1961 |
| depth_in_m | -0.05 | 0.01 | -0.07 | -0.04 | 1.00 | 1060 | 1789 |

**Family Specific Parameters**

|  | Estimate | Est.Error | l-95% CI | u-95% CI | Rhat | Bulk_ESS | Tail_ESS |
| --- | --- | --- | --- | --- | --- | --- | --- |
| shape | 14.38 | 0.38 | 13.66 | 15.15 | 1.00 | 5495 | 3407 |

**Table S4 | Summary tables of the Bayesian model for the weekly median of temperature range.** To test whether the weekly medians of temperature range vary significantly between regions (tropical, subtropical and temperate), we implemented a hierarchical modelling approach using Bayesian inference with Stan [(3)](https://www.zotero.org/google-docs/?5bTb50) and the “brms” package [(4)](https://www.zotero.org/google-docs/?q9s73K) within the R programming environment [(5)](https://www.zotero.org/google-docs/?dFYhHw). We specified models by ascribing variation among the data to “climate_classification” and “depth_in_m”, and grouped variation among geographically proximate locations to account for spatial autocorrelation by including a random intercept (“spatial_blocks”) for sampling sites falling within a 174 km radius of each other [(1)](https://www.zotero.org/google-docs/?kxfYfR). We used an additional level (“plot_id”) of random effects in a crossed design because the time series that were longer than 1 year in duration were divided into 1-year subsamples. Draws were sampled using sampling (NUTS). For each parameter, Bulk_ESS and Tail_ESS are effective sample size measures, and Rhat is the potential scale reduction factor on split chains (at convergence, Rhat = 1).

**Group-Level Effects**

| ~spatial_blocks (Number of levels: 120) | | | | | | | |
| --- | --- | --- | --- | --- | --- | --- | --- |
|  | Estimate | Est.Error | l-95% CI | u-95% CI | Rhat | Bulk_ESS | Tail_ESS |
| sd(Intercept) | 0.35 | 0.04 | 0.28 | 0.43 | 1.00 | 1106 | 2018 |
| ~spatial_blocks:plot_id (Number of levels: 487) | | | | | | | |
|  | Estimate | Est.Error | l-95% CI | u-95% CI | Rhat | Bulk_ESS | Tail_ESS |
| sd(Intercept) | 0.32 | 0.01 | 0.30 | 0.35 | 1.00 | 1147 | 1857 |

**Population-Level Effects**

|  | Estimate | Est.Error | l-95% CI | u-95% CI | Rhat | Bulk_ESS | Tail_ESS |
| --- | --- | --- | --- | --- | --- | --- | --- |
| Intercept | 0.86 | 0.07 | 0.71 | 1.00 | 1.00 | 1518 | 2158 |
| climate_classificationTemperate | -0.23 | 0.11 | -0.43 | -0.01 | 1.00 | 1492 | 1972 |
| climate_classificationTropical | -0.40 | 0.09 | -0.57 | -0.21 | 1.00 | 1558 | 2046 |
| depth_in_m | -0.05 | 0.01 | -0.06 | -0.04 | 1.00 | 1262 | 1962 |

**Family Specific Parameters**

|  | Estimate | Est.Error | l-95% CI | u-95% CI | Rhat | Bulk_ESS | Tail_ESS |
| --- | --- | --- | --- | --- | --- | --- | --- |
| shape | 17.72 | 0.47 | 16.80 | 18.65 | 1.00 | 4918 | 3068 |

**Table S5 | Summary tables of the Bayesian model for the bi-weekly median of temperature range.** To test whether the bi-weekly medians of temperature range vary significantly between regions (tropical, subtropical and temperate), we implemented a hierarchical modelling approach using Bayesian inference with Stan [(3)](https://www.zotero.org/google-docs/?0c8eUJ) and the “brms” package [(4)](https://www.zotero.org/google-docs/?iSZm8v) within the R programming environment [(5)](https://www.zotero.org/google-docs/?coQLiR). We specified models by ascribing variation among the data to “climate_classification” and “depth_in_m”, and grouped variation among geographically proximate locations to account for spatial autocorrelation by including a random intercept (“spatial_blocks”) for sampling sites falling within a 174 km radius of each other [(1)](https://www.zotero.org/google-docs/?N3s8pt). We used an additional level (“plot_id”) of random effects in a crossed design because the time series that were longer than 1 year in duration were divided into 1-year subsamples. Draws were sampled using sampling (NUTS). For each parameter, Bulk_ESS and Tail_ESS are effective sample size measures, and Rhat is the potential scale reduction factor on split chains (at convergence, Rhat = 1).

**Group-Level Effects**

| ~spatial_blocks (Number of levels: 120) | | | | | | | |
| --- | --- | --- | --- | --- | --- | --- | --- |
|  | Estimate | Est.Error | l-95% CI | u-95% CI | Rhat | Bulk_ESS | Tail_ESS |
| sd(Intercept) | 0.33 | 0.03 | 0.26 | 0.40 | 1.00 | 1128 | 2030 |
| ~spatial_blocks:plot_id (Number of levels: 487) | | | | | | | |
|  | Estimate | Est.Error | l-95% CI | u-95% CI | Rhat | Bulk_ESS | Tail_ESS |
| sd(Intercept) | 0.29 | 0.01 | 0.26 | 0.31 | 1.00 | 649 | 1848 |

**Population-Level Effects**

|  | Estimate | Est.Error | l-95% CI | u-95% CI | Rhat | Bulk_ESS | Tail_ESS |
| --- | --- | --- | --- | --- | --- | --- | --- |
| Intercept | 1.12 | 0.07 | 0.98 | 1.25 | 1.00 | 1607 | 2155 |
| climate_classificationTemperate | -0.18 | 0.10 | -0.37 | 0.01 | 1.00 | 1767 | 2163 |
| climate_classificationTropical | -0.39 | 0.08 | -0.55 | -0.24 | 1.00 | 1708 | 2474 |
| depth_in_m | -0.04 | 0.01 | -0.05 | -0.03 | 1.00 | 1637 | 2059 |

**Family Specific Parameters**

|  | Estimate | Est.Error | l-95% CI | u-95% CI | Rhat | Bulk_ESS | Tail_ESS |
| --- | --- | --- | --- | --- | --- | --- | --- |
| shape | 19.34 | 0.51 | 18.35 | 20.38 | 1.00 | 4773 | 2853 |

**Table S6 | Summary tables of the Bayesian model for the monthly median of temperature range.** To test whether the monthly medians of temperature range vary significantly between regions (tropical, subtropical and temperate), we implemented a hierarchical modelling approach using Bayesian inference with Stan [(3)](https://www.zotero.org/google-docs/?XomcRG) and the “brms” package [(4)](https://www.zotero.org/google-docs/?z8rnSE) within the R programming environment [(5)](https://www.zotero.org/google-docs/?SXKqmt). We specified models by ascribing variation among the data to “climate_classification” and “depth_in_m”, and grouped variation among geographically proximate locations to account for spatial autocorrelation by including a random intercept (“spatial_blocks”) for sampling sites falling within a 174 km radius of each other [(1)](https://www.zotero.org/google-docs/?Pp6jey). We used an additional level (“plot_id”) of random effects in a crossed design because the time series that were longer than 1 year in duration were divided into 1-year subsamples. Draws were sampled using sampling (NUTS). For each parameter, Bulk_ESS and Tail_ESS are effective sample size measures, and Rhat is the potential scale reduction factor on split chains (at convergence, Rhat = 1).

**Group-Level Effects**

| ~spatial_blocks (Number of levels: 120) | | | | | | | |
| --- | --- | --- | --- | --- | --- | --- | --- |
|  | Estimate | Est.Error | l-95% CI | u-95% CI | Rhat | Bulk_ESS | Tail_ESS |
| sd(Intercept) | 0.31 | 0.03 | 0.25 | 0.37 | 1.00 | 918 | 1335 |
| ~spatial_blocks:plot_id (Number of levels: 487) | | | | | | | |
|  | Estimate | Est.Error | l-95% CI | u-95% CI | Rhat | Bulk_ESS | Tail_ESS |
| sd(Intercept) | 0.25 | 0.01 | 0.23 | 0.27 | 1.00 | 917 | 2312 |

**Population-Level Effects**

|  | Estimate | Est.Error | l-95% CI | u-95% CI | Rhat | Bulk_ESS | Tail_ESS |
| --- | --- | --- | --- | --- | --- | --- | --- |
| Intercept | 1.37 | 0.06 | 1.25 | 1.50 | 1.00 | 1026 | 1540 |
| climate_classificationTemperate | -0.07 | 0.09 | -0.25 | 0.11 | 1.00 | 1147 | 1597 |
| climate_classificationTropical | -0.39 | 0.08 | -0.54 | -0.24 | 1.00 | 1022 | 1573 |
| depth_in_m | -0.04 | 0.00 | -0.05 | -0.03 | 1.00 | 1474 | 2412 |

**Family Specific Parameters**

|  | Estimate | Est.Error | l-95% CI | u-95% CI | Rhat | Bulk_ESS | Tail_ESS |
| --- | --- | --- | --- | --- | --- | --- | --- |
| shape | 20.42 | 0.55 | 19.39 | 21.53 | 1.00 | 5405 | 2889 |

**Table S7 | Summary tables of the Bayesian model for the annual median of temperature range.** To test whether the annual medians of temperature range vary significantly between regions (tropical, subtropical and temperate), we implemented a hierarchical modelling approach using Bayesian inference with Stan [(3)](https://www.zotero.org/google-docs/?pOoUf7) and the “brms” package [(4)](https://www.zotero.org/google-docs/?M31LGP) within the R programming environment [(5)](https://www.zotero.org/google-docs/?y0oc3F). We specified models by ascribing variation among the data to “climate_classification” and “depth_in_m”, and grouped variation among geographically proximate locations to account for spatial autocorrelation by including a random intercept (“spatial_blocks”) for sampling sites falling within a 174 km radius of each other [(1)](https://www.zotero.org/google-docs/?OXdU3H). Draws were sampled using sampling (NUTS). For each parameter, Bulk_ESS and Tail_ESS are effective sample size measures, and Rhat is the potential scale reduction factor on split chains (at convergence, Rhat = 1).

**Group-Level Effects**

| ~spatial_blocks (Number of levels: 120) | | | | | | | |
| --- | --- | --- | --- | --- | --- | --- | --- |
|  | Estimate | Est.Error | l-95% CI | u-95% CI | Rhat | Bulk_ESS | Tail_ESS |
| sd(Intercept) | 0.29 | 0.03 | 0.24 | 0.35 | 1.00 | 973 | 1817 |

**Population-Level Effects**

|  | Estimate | Est.Error | l-95% CI | u-95% CI | Rhat | Bulk_ESS | Tail_ESS |
| --- | --- | --- | --- | --- | --- | --- | --- |
| Intercept | 2.30 | 0.06 | 2.19 | 2.41 | 1.00 | 1026 | 1863 |
| climate_classificationTemperate | 0.22 | 0.08 | 0.06 | 0.38 | 1.00 | 1087 | 1613 |
| climate_classificationTropical | -0.46 | 0.06 | -0.59 | -0.34 | 1.00 | 1195 | 1885 |
| depth_in_m | -0.02 | 0.00 | -0.03 | -0.02 | 1.00 | 4287 | 3001 |

**Family Specific Parameters**

|  | Estimate | Est.Error | l-95% CI | u-95% CI | Rhat | Bulk_ESS | Tail_ESS |
| --- | --- | --- | --- | --- | --- | --- | --- |
| shape | 23.01 | 1.69 | 19.87 | 26.50 | 1.00 | 3473 | 3323 |

**Table S8 | Summary tables of the Bayesian model for the quarter-diurnal 90th percentile of temperature range.** To test whether the quarter-diurnal 90th percentile of temperature range vary significantly between regions (tropical, subtropical and temperate), we implemented a hierarchical modelling approach using Bayesian inference with Stan [(3)](https://www.zotero.org/google-docs/?AYIWK3) and the “brms” package [(4)](https://www.zotero.org/google-docs/?NCSrMM) within the R programming environment [(5)](https://www.zotero.org/google-docs/?CpzS3D). We specified models by ascribing variation among the data to “climate_classification” and “depth_in_m”, and grouped variation among geographically proximate locations to account for spatial autocorrelation by including a random intercept (“spatial_blocks”) for sampling sites falling within a 174 km radius of each other [(1)](https://www.zotero.org/google-docs/?sG06iU). We used an additional level (“plot_id”) of random effects in a crossed design because the time series that were longer than 1 year in duration were divided into 1-year subsamples. Draws were sampled using sampling (NUTS). For each parameter, Bulk_ESS and Tail_ESS are effective sample size measures, and Rhat is the potential scale reduction factor on split chains (at convergence, Rhat = 1).

**Group-Level Effects**

| ~spatial_blocks (Number of levels: 120) | | | | | | | |
| --- | --- | --- | --- | --- | --- | --- | --- |
|  | Estimate | Est.Error | l-95% CI | u-95% CI | Rhat | Bulk_ESS | Tail_ESS |
| sd(Intercept) | 0.42 | 0.05 | 0.33 | 0.52 | 1.00 | 877 | 1577 |
| ~spatial_blocks:plot_id (Number of levels: 487) | | | | | | | |
|  | Estimate | Est.Error | l-95% CI | u-95% CI | Rhat | Bulk_ESS | Tail_ESS |
| sd(Intercept) | 0.42 | 0.02 | 0.38 | 0.45 | 1.00 | 953 | 1510 |

**Population-Level Effects**

|  | Estimate | Est.Error | l-95% CI | u-95% CI | Rhat | Bulk_ESS | Tail_ESS |
| --- | --- | --- | --- | --- | --- | --- | --- |
| Intercept | -0.03 | 0.09 | -0.21 | 0.15 | 1.00 | 1094 | 1762 |
| climate_classificationTemperate | -0.14 | 0.13 | -0.40 | 0.11 | 1.00 | 1022 | 1595 |
| climate_classificationTropical | -0.35 | 0.11 | -0.57 | -0.13 | 1.00 | 1220 | 1933 |
| depth_in_m | -0.03 | 0.01 | -0.05 | -0.01 | 1.00 | 850 | 1415 |

**Family Specific Parameters**

|  | Estimate | Est.Error | l-95% CI | u-95% CI | Rhat | Bulk_ESS | Tail_ESS |
| --- | --- | --- | --- | --- | --- | --- | --- |
| shape | 14.05 | 0.37 | 13.33 | 14.78 | 1.00 | 5532 | 3043 |

**Table S9 | Summary tables of the Bayesian model for the semi-diurnal 90th percentile of temperature range.** To test whether the semi-diurnal 90th percentile of temperature range vary significantly between regions (tropical, subtropical and temperate), we implemented a hierarchical modelling approach using Bayesian inference with Stan [(3)](https://www.zotero.org/google-docs/?oz44kz) and the “brms” package [(4)](https://www.zotero.org/google-docs/?Tz0fru) within the R programming environment [(5)](https://www.zotero.org/google-docs/?y6eVma). We specified models by ascribing variation among the data to “climate_classification” and “depth_in_m”, and grouped variation among geographically proximate locations to account for spatial autocorrelation by including a random intercept (“spatial_blocks”) for sampling sites falling within a 174 km radius of each other [(1)](https://www.zotero.org/google-docs/?9ewNE3). We used an additional level (“plot_id”) of random effects in a crossed design because the time series that were longer than 1 year in duration were divided into 1-year subsamples. Draws were sampled using sampling (NUTS). For each parameter, Bulk_ESS and Tail_ESS are effective sample size measures, and Rhat is the potential scale reduction factor on split chains (at convergence, Rhat = 1).

**Group-Level Effects**

| ~spatial_blocks (Number of levels: 120) | | | | | | | |
| --- | --- | --- | --- | --- | --- | --- | --- |
|  | Estimate | Est.Error | l-95% CI | u-95% CI | Rhat | Bulk_ESS | Tail_ESS |
| sd(Intercept) | 0.40 | 0.04 | 0.32 | 0.50 | 1.00 | 837 | 1771 |
| ~spatial_blocks:plot_id (Number of levels: 487) | | | | | | | |
|  | Estimate | Est.Error | l-95% CI | u-95% CI | Rhat | Bulk_ESS | Tail_ESS |
| sd(Intercept) | 0.40 | 0.02 | 0.37 | 0.44 | 1.00 | 909 | 1676 |

**Population-Level Effects**

|  | Estimate | Est.Error | l-95% CI | u-95% CI | Rhat | Bulk_ESS | Tail_ESS |
| --- | --- | --- | --- | --- | --- | --- | --- |
| Intercept | 0.37 | 0.09 | 0.20 | 0.54 | 1.00 | 1134 | 1710 |
| climate_classificationTemperate | -0.10 | 0.12 | -0.33 | 0.16 | 1.00 | 1067 | 1616 |
| climate_classificationTropical | -0.35 | 0.10 | -0.55 | -0.13 | 1.00 | 1272 | 1950 |
| depth_in_m | -0.04 | 0.01 | -0.05 | -0.02 | 1.00 | 905 | 1296 |

**Family Specific Parameters**

|  | Estimate | Est.Error | l-95% CI | u-95% CI | Rhat | Bulk_ESS | Tail_ESS |
| --- | --- | --- | --- | --- | --- | --- | --- |
| shape | 14.34 | 0.39 | 13.59 | 15.13 | 1.00 | 4505 | 2540 |

**Table S10 | Summary tables of the Bayesian model for the diurnal 90th percentile of temperature range.** To test whether the diurnal 90th percentile of temperature range vary significantly between regions (tropical, subtropical and temperate), we implemented a hierarchical modelling approach using Bayesian inference with Stan [(3)](https://www.zotero.org/google-docs/?GSESby) and the “brms” package [(4)](https://www.zotero.org/google-docs/?gybeld) within the R programming environment [(5)](https://www.zotero.org/google-docs/?uLCFY1). We specified models by ascribing variation among the data to “climate_classification” and “depth_in_m”, and grouped variation among geographically proximate locations to account for spatial autocorrelation by including a random intercept (“spatial_blocks”) for sampling sites falling within a 174 km radius of each other [(1)](https://www.zotero.org/google-docs/?50roTL). We used an additional level (“plot_id”) of random effects in a crossed design because the time series that were longer than 1 year in duration were divided into 1-year subsamples. Draws were sampled using sampling (NUTS). For each parameter, Bulk_ESS and Tail_ESS are effective sample size measures, and Rhat is the potential scale reduction factor on split chains (at convergence, Rhat = 1).

**Group-Level Effects**

| ~spatial_blocks (Number of levels: 120) | | | | | | | |
| --- | --- | --- | --- | --- | --- | --- | --- |
|  | Estimate | Est.Error | l-95% CI | u-95% CI | Rhat | Bulk_ESS | Tail_ESS |
| sd(Intercept) | 0.39 | 0.04 | 0.31 | 0.48 | 1.00 | 933 | 1529 |
| ~spatial_blocks:plot_id (Number of levels: 487) | | | | | | | |
|  | Estimate | Est.Error | l-95% CI | u-95% CI | Rhat | Bulk_ESS | Tail_ESS |
| sd(Intercept) | 0.39 | 0.02 | 0.36 | 0.42 | 1.01 | 965 | 1190 |

**Population-Level Effects**

|  | Estimate | Est.Error | l-95% CI | u-95% CI | Rhat | Bulk_ESS | Tail_ESS |
| --- | --- | --- | --- | --- | --- | --- | --- |
| Intercept | 0.72 | 0.08 | 0.56 | 0.88 | 1.00 | 1357 | 2160 |
| climate_classificationTemperate | -0.07 | 0.12 | -0.31 | 0.16 | 1.00 | 1381 | 1961 |
| climate_classificationTropical | -0.38 | 0.10 | -0.57 | -0.18 | 1.00 | 1421 | 1996 |
| depth_in_m | -0.04 | 0.01 | -0.06 | -0.03 | 1.00 | 1033 | 1730 |

**Family Specific Parameters**

|  | Estimate | Est.Error | l-95% CI | u-95% CI | Rhat | Bulk_ESS | Tail_ESS |
| --- | --- | --- | --- | --- | --- | --- | --- |
| shape | 14.99 | 0.40 | 14.18 | 15.79 | 1.00 | 5776 | 2712 |

**Table S11 | Summary tables of the Bayesian model for the weekly 90th percentile of temperature range.** To test whether the weekly 90th percentile of temperature range vary significantly between regions (tropical, subtropical and temperate), we implemented a hierarchical modelling approach using Bayesian inference with Stan [(3)](https://www.zotero.org/google-docs/?OqkthJ) and the “brms” package [(4)](https://www.zotero.org/google-docs/?gOpi09) within the R programming environment [(5)](https://www.zotero.org/google-docs/?VA32ZW). We specified models by ascribing variation among the data to “climate_classification” and “depth_in_m”, and grouped variation among geographically proximate locations to account for spatial autocorrelation by including a random intercept (“spatial_blocks”) for sampling sites falling within a 174 km radius of each other [(1)](https://www.zotero.org/google-docs/?aOD2dV). We used an additional level (“plot_id”) of random effects in a crossed design because the time series that were longer than 1 year in duration were divided into 1-year subsamples. Draws were sampled using sampling (NUTS). For each parameter, Bulk_ESS and Tail_ESS are effective sample size measures, and Rhat is the potential scale reduction factor on split chains (at convergence, Rhat = 1).

**Group-Level Effects**

| ~spatial_blocks (Number of levels: 120) | | | | | | | |
| --- | --- | --- | --- | --- | --- | --- | --- |
|  | Estimate | Est.Error | l-95% CI | u-95% CI | Rhat | Bulk_ESS | Tail_ESS |
| sd(Intercept) | 0.38 | 0.04 | 0.31 | 0.46 | 1.00 | 1208 | 1745 |
| ~spatial_blocks:plot_id (Number of levels: 487) | | | | | | | |
|  | Estimate | Est.Error | l-95% CI | u-95% CI | Rhat | Bulk_ESS | Tail_ESS |
| sd(Intercept) | 0.29 | 0.01 | 0.27 | 0.32 | 1.00 | 1099 | 1903 |

**Population-Level Effects**

|  | Estimate | Est.Error | l-95% CI | u-95% CI | Rhat | Bulk_ESS | Tail_ESS |
| --- | --- | --- | --- | --- | --- | --- | --- |
| Intercept | 1.31 | 0.08 | 1.16 | 1.46 | 1.00 | 1361 | 1932 |
| climate_classificationTemperate | 0.04 | 0.11 | -0.17 | 0.26 | 1.00 | 1334 | 1935 |
| climate_classificationTropical | -0.37 | 0.09 | -0.55 | -0.18 | 1.00 | 1246 | 1679 |
| depth_in_m | -0.04 | 0.01 | -0.05 | -0.03 | 1.00 | 1383 | 1972 |

**Family Specific Parameters**

|  | Estimate | Est.Error | l-95% CI | u-95% CI | Rhat | Bulk_ESS | Tail_ESS |
| --- | --- | --- | --- | --- | --- | --- | --- |
| shape | 17.85 | 0.47 | 16.94 | 18.80 | 1.00 | 5897 | 3011 |

**Table S12 | Summary tables of the Bayesian model for the bi-weekly 90th percentile of temperature range.** To test whether the bi-weekly 90th percentile of temperature range vary significantly between regions (tropical, subtropical and temperate), we implemented a hierarchical modelling approach using Bayesian inference with Stan [(3)](https://www.zotero.org/google-docs/?1C1XwB) and the “brms” package [(4)](https://www.zotero.org/google-docs/?ZNBw1b) within the R programming environment [(5)](https://www.zotero.org/google-docs/?1cZ0rx). We specified models by ascribing variation among the data to “climate_classification” and “depth_in_m”, and grouped variation among geographically proximate locations to account for spatial autocorrelation by including a random intercept (“spatial_blocks”) for sampling sites falling within a 174 km radius of each other [(1)](https://www.zotero.org/google-docs/?Bkry4T). We used an additional level (“plot_id”) of random effects in a crossed design because the time series that were longer than 1 year in duration were divided into 1-year subsamples. Draws were sampled using sampling (NUTS). For each parameter, Bulk_ESS and Tail_ESS are effective sample size measures, and Rhat is the potential scale reduction factor on split chains (at convergence, Rhat = 1).

**Group-Level Effects**

| ~spatial_blocks (Number of levels: 120) | | | | | | | |
| --- | --- | --- | --- | --- | --- | --- | --- |
|  | Estimate | Est.Error | l-95% CI | u-95% CI | Rhat | Bulk_ESS | Tail_ESS |
| sd(Intercept) | 0.36 | 0.03 | 0.30 | 0.42 | 1.00 | 1313 | 1783 |
| ~spatial_blocks:plot_id (Number of levels: 487) | | | | | | | |
|  | Estimate | Est.Error | l-95% CI | u-95% CI | Rhat | Bulk_ESS | Tail_ESS |
| sd(Intercept) | 0.26 | 0.01 | 0.24 | 0.28 | 1.00 | 1248 | 2277 |

**Population-Level Effects**

|  | Estimate | Est.Error | l-95% CI | u-95% CI | Rhat | Bulk_ESS | Tail_ESS |
| --- | --- | --- | --- | --- | --- | --- | --- |
| Intercept | 1.50 | 0.07 | 1.36 | 1.63 | 1.00 | 1186 | 1691 |
| climate_classificationTemperate | 0.07 | 0.10 | -0.11 | 0.27 | 1.00 | 1201 | 1788 |
| climate_classificationTropical | -0.38 | 0.08 | -0.55 | -0.22 | 1.00 | 1209 | 2016 |
| depth_in_m | -0.03 | 0.01 | -0.04 | -0.02 | 1.00 | 1488 | 2115 |

**Family Specific Parameters**

|  | Estimate | Est.Error | l-95% CI | u-95% CI | Rhat | Bulk_ESS | Tail_ESS |
| --- | --- | --- | --- | --- | --- | --- | --- |
| shape | 19.03 | 0.51 | 18.07 | 20.06 | 1.00 | 4663 | 2994 |

**Table S13 | Summary tables of the Bayesian model for the monthly 90th percentile of temperature range.** To test whether the monthly 90th percentile of temperature range vary significantly between regions (tropical, subtropical and temperate), we implemented a hierarchical modelling approach using Bayesian inference with Stan [(3)](https://www.zotero.org/google-docs/?ra6s7R) and the “brms” package [(4)](https://www.zotero.org/google-docs/?K8gFeO) within the R programming environment [(5)](https://www.zotero.org/google-docs/?9DdAue). We specified models by ascribing variation among the data to “climate_classification” and “depth_in_m”, and grouped variation among geographically proximate locations to account for spatial autocorrelation by including a random intercept (“spatial_blocks”) for sampling sites falling within a 174 km radius of each other [(1)](https://www.zotero.org/google-docs/?S3jfyp). We used an additional level (“plot_id”) of random effects in a crossed design because the time series that were longer than 1 year in duration were divided into 1-year subsamples. Draws were sampled using sampling (NUTS). For each parameter, Bulk_ESS and Tail_ESS are effective sample size measures, and Rhat is the potential scale reduction factor on split chains (at convergence, Rhat = 1).

**Group-Level Effects**

| ~spatial_blocks (Number of levels: 120) | | | | | | | |
| --- | --- | --- | --- | --- | --- | --- | --- |
|  | Estimate | Est.Error | l-95% CI | u-95% CI | Rhat | Bulk_ESS | Tail_ESS |
| sd(Intercept) | 0.33 | 0.03 | 0.28 | 0.40 | 1.00 | 1597 | 2396 |
| ~spatial_blocks:plot_id (Number of levels: 487) | | | | | | | |
|  | Estimate | Est.Error | l-95% CI | u-95% CI | Rhat | Bulk_ESS | Tail_ESS |
| sd(Intercept) | 0.22 | 0.01 | 0.20 | 0.24 | 1.00 | 1299 | 1966 |

**Population-Level Effects**

|  | Estimate | Est.Error | l-95% CI | u-95% CI | Rhat | Bulk_ESS | Tail_ESS |
| --- | --- | --- | --- | --- | --- | --- | --- |
| Intercept | 1.69 | 0.06 | 1.56 | 1.82 | 1.00 | 1615 | 1982 |
| climate_classificationTemperate | 0.10 | 0.09 | -0.07 | 0.28 | 1.00 | 1485 | 2327 |
| climate_classificationTropical | -0.40 | 0.08 | -0.55 | -0.25 | 1.00 | 1886 | 2688 |
| depth_in_m | -0.03 | 0.00 | -0.04 | -0.02 | 1.00 | 2309 | 2658 |

**Family Specific Parameters**

|  | Estimate | Est.Error | l-95% CI | u-95% CI | Rhat | Bulk_ESS | Tail_ESS |
| --- | --- | --- | --- | --- | --- | --- | --- |
| shape | 19.66 | 0.54 | 18.64 | 20.73 | 1.00 | 5544 | 2878 |

**Table S14 | Summary tables of the Bayesian model for the annual 90th percentile of temperature range.** To test whether the annual 90th percentile of temperature range vary significantly between regions (tropical, subtropical and temperate), we implemented a hierarchical modelling approach using Bayesian inference with Stan [(3)](https://www.zotero.org/google-docs/?v2DaQQ) and the “brms” package [(4)](https://www.zotero.org/google-docs/?5xG9X0) within the R programming environment [(5)](https://www.zotero.org/google-docs/?gbpqIy). We specified models by ascribing variation among the data to “climate_classification” and “depth_in_m”, and grouped variation among geographically proximate locations to account for spatial autocorrelation by including a random intercept (“spatial_blocks”) for sampling sites falling within a 174 km radius of each other [(1)](https://www.zotero.org/google-docs/?xoKRfb). Draws were sampled using sampling (NUTS). For each parameter, Bulk_ESS and Tail_ESS are effective sample size measures, and Rhat is the potential scale reduction factor on split chains (at convergence, Rhat = 1).

**Group-Level Effects**

| ~spatial_blocks (Number of levels: 120) | | | | | | | |
| --- | --- | --- | --- | --- | --- | --- | --- |
|  | Estimate | Est.Error | l-95% CI | u-95% CI | Rhat | Bulk_ESS | Tail_ESS |
| sd(Intercept) | 0.30 | 0.03 | 0.25 | 0.35 | 1.00 | 705 | 1403 |

**Population-Level Effects**

|  | Estimate | Est.Error | l-95% CI | u-95% CI | Rhat | Bulk_ESS | Tail_ESS |
| --- | --- | --- | --- | --- | --- | --- | --- |
| Intercept | 2.39 | 0.05 | 2.28 | 2.49 | 1.01 | 990 | 1272 |
| climate_classificationTemperate | 0.24 | 0.08 | 0.08 | 0.39 | 1.00 | 849 | 1364 |
| climate_classificationTropical | -0.39 | 0.06 | -0.51 | -0.27 | 1.00 | 1059 | 1377 |
| depth_in_m | -0.02 | 0.00 | -0.03 | -0.01 | 1.01 | 4562 | 3429 |

**Family Specific Parameters**

|  | Estimate | Est.Error | l-95% CI | u-95% CI | Rhat | Bulk_ESS | Tail_ESS |
| --- | --- | --- | --- | --- | --- | --- | --- |
| shape | 27.25 | 2.07 | 23.36 | 31.56 | 1.00 | 2792 | 2530 |

**Table S15 | Summary tables of the Bayesian model for the quarter-diurnal median of difference in biological rates.** To test whether the quarter-diurnal median of difference in biological rates vary significantly between regions (tropical, subtropical and temperate), we implemented a hierarchical modelling approach using Bayesian inference with Stan [(3)](https://www.zotero.org/google-docs/?YkeKe2) and the “brms” package [(4)](https://www.zotero.org/google-docs/?5Zp5cy) within the R programming environment [(5)](https://www.zotero.org/google-docs/?8OFUQK). We specified models by ascribing variation among the data to “climate_classification” and “depth_in_m”, and grouped variation among geographically proximate locations to account for spatial autocorrelation by including a random intercept (“spatial_blocks”) for sampling sites falling within a 174 km radius of each other [(1)](https://www.zotero.org/google-docs/?N5LBgP). We used an additional level (“plot_id”) of random effects in a crossed design because the time series that were longer than 1 year in duration were divided into 1-year subsamples. Draws were sampled using sampling (NUTS). For each parameter, Bulk_ESS and Tail_ESS are effective sample size measures, and Rhat is the potential scale reduction factor on split chains (at convergence, Rhat = 1).

**Group-Level Effects**

| ~spatial_blocks (Number of levels: 120) | | | | | | | |
| --- | --- | --- | --- | --- | --- | --- | --- |
|  | Estimate | Est.Error | l-95% CI | u-95% CI | Rhat | Bulk_ESS | Tail_ESS |
| sd(Intercept) | 0.50 | 0.05 | 0.40 | 0.61 | 1.00 | 936 | 1633 |
| ~spatial_blocks:plot_id (Number of levels: 486) | | | | | | | |
|  | Estimate | Est.Error | l-95% CI | u-95% CI | Rhat | Bulk_ESS | Tail_ESS |
| sd(Intercept) | 0.44 | 0.02 | 0.40 | 0.48 | 1.00 | 881 | 1489 |

**Population-Level Effects**

|  | Estimate | Est.Error | l-95% CI | u-95% CI | Rhat | Bulk_ESS | Tail_ESS |
| --- | --- | --- | --- | --- | --- | --- | --- |
| Intercept | -17.90 | 0.10 | -18.11 | -17.70 | 1.00 | 1167 | 1871 |
| climate_classificationTemperate | -1.11 | 0.15 | -1.40 | -0.82 | 1.00 | 1058 | 1407 |
| climate_classificationTropical | 0.06 | 0.12 | -0.19 | 0.30 | 1.00 | 1213 | 2115 |
| depth_in_m | -0.04 | 0.01 | -0.06 | -0.02 | 1.00 | 799 | 1224 |

**Family Specific Parameters**

|  | Estimate | Est.Error | l-95% CI | u-95% CI | Rhat | Bulk_ESS | Tail_ESS |
| --- | --- | --- | --- | --- | --- | --- | --- |
| shape | 10.54 | 0.29 | 9.98 | 11.13 | 1.00 | 4920 | 2669 |

**Table S16 | Summary tables of the Bayesian model for the semi-diurnal median of difference in biological rates.** To test whether the semi-diurnal median of difference in biological rates vary significantly between regions (tropical, subtropical and temperate), we implemented a hierarchical modelling approach using Bayesian inference with Stan [(3)](https://www.zotero.org/google-docs/?WocsfA) and the “brms” package [(4)](https://www.zotero.org/google-docs/?KZKD2F) within the R programming environment [(5)](https://www.zotero.org/google-docs/?zdpRXG). We specified models by ascribing variation among the data to “climate_classification” and “depth_in_m”, and grouped variation among geographically proximate locations to account for spatial autocorrelation by including a random intercept (“spatial_blocks”) for sampling sites falling within a 174 km radius of each other [(1)](https://www.zotero.org/google-docs/?Eobt2c). We used an additional level (“plot_id”) of random effects in a crossed design because the time series that were longer than 1 year in duration were divided into 1-year subsamples. Draws were sampled using sampling (NUTS). For each parameter, Bulk_ESS and Tail_ESS are effective sample size measures, and Rhat is the potential scale reduction factor on split chains (at convergence, Rhat = 1).

**Group-Level Effects**

| ~spatial_blocks (Number of levels: 120) | | | | | | | |
| --- | --- | --- | --- | --- | --- | --- | --- |
|  | Estimate | Est.Error | l-95% CI | u-95% CI | Rhat | Bulk_ESS | Tail_ESS |
| sd(Intercept) | 0.46 | 0.05 | 0.37 | 0.57 | 1.00 | 1324 | 1942 |
| ~spatial_blocks:plot_id (Number of levels: 487) | | | | | | | |
|  | Estimate | Est.Error | l-95% CI | u-95% CI | Rhat | Bulk_ESS | Tail_ESS |
| sd(Intercept) | 0.44 | 0.02 | 0.40 | 0.48 | 1.00 | 1178 | 2004 |

**Population-Level Effects**

|  | Estimate | Est.Error | l-95% CI | u-95% CI | Rhat | Bulk_ESS | Tail_ESS |
| --- | --- | --- | --- | --- | --- | --- | --- |
| Intercept | -17.30 | 0.10 | -17.51 | -17.10 | 1.00 | 2857 | 2629 |
| climate_classificationTemperate | -1.11 | 0.14 | -1.38 | -0.85 | 1.00 | 2924 | 2694 |
| climate_classificationTropical | 0.06 | 0.12 | -0.17 | 0.29 | 1.00 | 3069 | 2675 |
| depth_in_m | -0.05 | 0.01 | -0.06 | -0.03 | 1.00 | 2512 | 2603 |

**Family Specific Parameters**

|  | Estimate | Est.Error | l-95% CI | u-95% CI | Rhat | Bulk_ESS | Tail_ESS |
| --- | --- | --- | --- | --- | --- | --- | --- |
| shape | 10.55 | 0.28 | 10.01 | 11.08 | 1.00 | 4058 | 2816 |

**Table S17 | Summary tables of the Bayesian model for the diurnal median of difference in biological rates.** To test whether the diurnal median of difference in biological rates vary significantly between regions (tropical, subtropical and temperate), we implemented a hierarchical modelling approach using Bayesian inference with Stan [(3)](https://www.zotero.org/google-docs/?eeb2Iu) and the “brms” package [(4)](https://www.zotero.org/google-docs/?EKdEvo) within the R programming environment [(5)](https://www.zotero.org/google-docs/?OrOS02). We specified models by ascribing variation among the data to “climate_classification” and “depth_in_m”, and grouped variation among geographically proximate locations to account for spatial autocorrelation by including a random intercept (“spatial_blocks”) for sampling sites falling within a 174 km radius of each other [(1)](https://www.zotero.org/google-docs/?fm1gDq). We used an additional level (“plot_id”) of random effects in a crossed design because the time series that were longer than 1 year in duration were divided into 1-year subsamples. Draws were sampled using sampling (NUTS). For each parameter, Bulk_ESS and Tail_ESS are effective sample size measures, and Rhat is the potential scale reduction factor on split chains (at convergence, Rhat = 1).

**Group-Level Effects**

| ~spatial_blocks (Number of levels: 120) | | | | | | | |
| --- | --- | --- | --- | --- | --- | --- | --- |
|  | Estimate | Est.Error | l-95% CI | u-95% CI | Rhat | Bulk_ESS | Tail_ESS |
| sd(Intercept) | 0.46 | 0.05 | 0.37 | 0.56 | 1.00 | 1115 | 1876 |
| ~spatial_blocks:plot_id (Number of levels: 487) | | | | | | | |
|  | Estimate | Est.Error | l-95% CI | u-95% CI | Rhat | Bulk_ESS | Tail_ESS |
| sd(Intercept) | 0.42 | 0.02 | 0.39 | 0.46 | 1.00 | 980 | 1693 |

**Population-Level Effects**

|  | Estimate | Est.Error | l-95% CI | u-95% CI | Rhat | Bulk_ESS | Tail_ESS |
| --- | --- | --- | --- | --- | --- | --- | --- |
| Intercept | -16.84 | 0.10 | -17.03 | -16.65 | 1.01 | 1054 | 1721 |
| climate_classificationTemperate | -1.10 | 0.14 | -1.37 | -0.84 | 1.00 | 1300 | 1959 |
| climate_classificationTropical | -0.01 | 0.11 | -0.23 | 0.21 | 1.00 | 1161 | 2009 |
| depth_in_m | -0.05 | 0.01 | -0.07 | -0.04 | 1.00 | 974 | 1683 |

**Family Specific Parameters**

|  | Estimate | Est.Error | l-95% CI | u-95% CI | Rhat | Bulk_ESS | Tail_ESS |
| --- | --- | --- | --- | --- | --- | --- | --- |
| shape | 11.20 | 0.28 | 10.66 | 11.74 | 1.00 | 5433 | 3195 |

**Table S18 | Summary tables of the Bayesian model for the weekly median of difference in biological rates.** To test whether the weekly median of difference in biological rates vary significantly between regions (tropical, subtropical and temperate), we implemented a hierarchical modelling approach using Bayesian inference with Stan [(3)](https://www.zotero.org/google-docs/?XJYCXk) and the “brms” package [(4)](https://www.zotero.org/google-docs/?6W52na) within the R programming environment [(5)](https://www.zotero.org/google-docs/?mEP9hy). We specified models by ascribing variation among the data to “climate_classification” and “depth_in_m”, and grouped variation among geographically proximate locations to account for spatial autocorrelation by including a random intercept (“spatial_blocks”) for sampling sites falling within a 174 km radius of each other [(1)](https://www.zotero.org/google-docs/?pdBDQo). We used an additional level (“plot_id”) of random effects in a crossed design because the time series that were longer than 1 year in duration were divided into 1-year subsamples. Draws were sampled using sampling (NUTS). For each parameter, Bulk_ESS and Tail_ESS are effective sample size measures, and Rhat is the potential scale reduction factor on split chains (at convergence, Rhat = 1).

**Group-Level Effects**

| ~spatial_blocks (Number of levels: 120) | | | | | | | |
| --- | --- | --- | --- | --- | --- | --- | --- |
|  | Estimate | Est.Error | l-95% CI | u-95% CI | Rhat | Bulk_ESS | Tail_ESS |
| sd(Intercept) | 0.39 | 0.04 | 0.32 | 0.48 | 1.01 | 991 | 1560 |
| ~spatial_blocks:plot_id (Number of levels: 487) | | | | | | | |
|  | Estimate | Est.Error | l-95% CI | u-95% CI | Rhat | Bulk_ESS | Tail_ESS |
| sd(Intercept) | 0.33 | 0.01 | 0.30 | 0.36 | 1.00 | 893 | 2420 |

**Population-Level Effects**

|  | Estimate | Est.Error | l-95% CI | u-95% CI | Rhat | Bulk_ESS | Tail_ESS |
| --- | --- | --- | --- | --- | --- | --- | --- |
| Intercept | -16.04 | 0.08 | -16.20 | -15.88 | 1.00 | 1024 | 1682 |
| climate_classificationTemperate | -0.93 | 0.12 | -1.15 | -0.71 | 1.00 | 1214 | 1954 |
| climate_classificationTropical | 0.03 | 0.10 | -0.17 | 0.22 | 1.00 | 1183 | 1534 |
| depth_in_m | -0.05 | 0.01 | -0.06 | -0.03 | 1.00 | 987 | 1855 |

**Family Specific Parameters**

|  | Estimate | Est.Error | l-95% CI | u-95% CI | Rhat | Bulk_ESS | Tail_ESS |
| --- | --- | --- | --- | --- | --- | --- | --- |
| shape | 12.98 | 0.35 | 12.31 | 13.69 | 1.00 | 5385 | 3020 |

**Table S19 | Summary tables of the Bayesian model for the bi-weekly median of difference in biological rates.** To test whether the bi-weekly median of difference in biological rates vary significantly between regions (tropical, subtropical and temperate), we implemented a hierarchical modelling approach using Bayesian inference with Stan [(3)](https://www.zotero.org/google-docs/?qrvY2k) and the “brms” package [(4)](https://www.zotero.org/google-docs/?K0HWi5) within the R programming environment [(5)](https://www.zotero.org/google-docs/?1Z1hoi). We specified models by ascribing variation among the data to “climate_classification” and “depth_in_m”, and grouped variation among geographically proximate locations to account for spatial autocorrelation by including a random intercept (“spatial_blocks”) for sampling sites falling within a 174 km radius of each other [(1)](https://www.zotero.org/google-docs/?RIVTks). We used an additional level (“plot_id”) of random effects in a crossed design because the time series that were longer than 1 year in duration were divided into 1-year subsamples. Draws were sampled using sampling (NUTS). For each parameter, Bulk_ESS and Tail_ESS are effective sample size measures, and Rhat is the potential scale reduction factor on split chains (at convergence, Rhat = 1).

**Group-Level Effects**

| ~spatial_blocks (Number of levels: 120) | | | | | | | |
| --- | --- | --- | --- | --- | --- | --- | --- |
|  | Estimate | Est.Error | l-95% CI | u-95% CI | Rhat | Bulk_ESS | Tail_ESS |
| sd(Intercept) | 0.38 | 0.04 | 0.31 | 0.46 | 1.00 | 1363 | 1898 |
| ~spatial_blocks:plot_id (Number of levels: 487) | | | | | | | |
|  | Estimate | Est.Error | l-95% CI | u-95% CI | Rhat | Bulk_ESS | Tail_ESS |
| sd(Intercept) | 0.29 | 0.01 | 0.27 | 0.32 | 1.00 | 1182 | 2050 |

**Population-Level Effects**

|  | Estimate | Est.Error | l-95% CI | u-95% CI | Rhat | Bulk_ESS | Tail_ESS |
| --- | --- | --- | --- | --- | --- | --- | --- |
| Intercept | -15.78 | 0.08 | -15.93 | -15.63 | 1.00 | 1422 | 1840 |
| climate_classificationTemperate | -0.87 | 0.11 | -1.09 | -0.65 | 1.00 | 1272 | 1778 |
| climate_classificationTropical | 0.04 | 0.09 | -0.13 | 0.22 | 1.00 | 1579 | 2141 |
| depth_in_m | -0.04 | 0.01 | -0.06 | -0.03 | 1.00 | 1240 | 1883 |

**Family Specific Parameters**

|  | Estimate | Est.Error | l-95% CI | u-95% CI | Rhat | Bulk_ESS | Tail_ESS |
| --- | --- | --- | --- | --- | --- | --- | --- |
| shape | 13.75 | 0.36 | 13.04 | 14.47 | 1.00 | 4696 | 3013 |

**Table S20 | Summary tables of the Bayesian model for the monthly median of difference in biological rates.** To test whether the monthly median of difference in biological rates vary significantly between regions (tropical, subtropical and temperate), we implemented a hierarchical modelling approach using Bayesian inference with Stan [(3)](https://www.zotero.org/google-docs/?4ZwfVF) and the “brms” package [(4)](https://www.zotero.org/google-docs/?mLCQSC) within the R programming environment [(5)](https://www.zotero.org/google-docs/?wIr4c4). We specified models by ascribing variation among the data to “climate_classification” and “depth_in_m”, and grouped variation among geographically proximate locations to account for spatial autocorrelation by including a random intercept (“spatial_blocks”) for sampling sites falling within a 174 km radius of each other [(1)](https://www.zotero.org/google-docs/?AvPrli). We used an additional level (“plot_id”) of random effects in a crossed design because the time series that were longer than 1 year in duration were divided into 1-year subsamples. Draws were sampled using sampling (NUTS). For each parameter, Bulk_ESS and Tail_ESS are effective sample size measures, and Rhat is the potential scale reduction factor on split chains (at convergence, Rhat = 1).

**Group-Level Effects**

| ~spatial_blocks (Number of levels: 120) | | | | | | | |
| --- | --- | --- | --- | --- | --- | --- | --- |
|  | Estimate | Est.Error | l-95% CI | u-95% CI | Rhat | Bulk_ESS | Tail_ESS |
| sd(Intercept) | 0.38 | 0.03 | 0.32 | 0.45 | 1.00 | 1346 | 2128 |
| ~spatial_blocks:plot_id (Number of levels: 487) | | | | | | | |
|  | Estimate | Est.Error | l-95% CI | u-95% CI | Rhat | Bulk_ESS | Tail_ESS |
| sd(Intercept) | 0.25 | 0.01 | 0.23 | 0.28 | 1.00 | 1166 | 2017 |

**Population-Level Effects**

|  | Estimate | Est.Error | l-95% CI | u-95% CI | Rhat | Bulk_ESS | Tail_ESS |
| --- | --- | --- | --- | --- | --- | --- | --- |
| Intercept | -15.50 | 0.07 | -15.65 | -15.36 | 1.00 | 968 | 1484 |
| climate_classificationTemperate | -0.74 | 0.11 | -0.94 | -0.54 | 1.00 | 982 | 1710 |
| climate_classificationTropical | 0.04 | 0.09 | -0.13 | 0.21 | 1.00 | 845 | 1500 |
| depth_in_m | -0.04 | 0.01 | -0.05 | -0.03 | 1.00 | 1525 | 2542 |

**Family Specific Parameters**

|  | Estimate | Est.Error | l-95% CI | u-95% CI | Rhat | Bulk_ESS | Tail_ESS |
| --- | --- | --- | --- | --- | --- | --- | --- |
| shape | 14.67 | 0.40 | 13.88 | 15.46 | 1.00 | 5059 | 2963 |

**Table S21 | Summary tables of the Bayesian model for the annual median of difference in biological rates.** To test whether the annual median of difference in biological rates vary significantly between regions (tropical, subtropical and temperate), we implemented a hierarchical modelling approach using Bayesian inference with Stan [(3)](https://www.zotero.org/google-docs/?d22z2b) and the “brms” package [(4)](https://www.zotero.org/google-docs/?UzeBsF) within the R programming environment [(5)](https://www.zotero.org/google-docs/?pYF6fN). We specified models by ascribing variation among the data to “climate_classification” and “depth_in_m”, and grouped variation among geographically proximate locations to account for spatial autocorrelation by including a random intercept (“spatial_blocks”) for sampling sites falling within a 174 km radius of each other [(1)](https://www.zotero.org/google-docs/?yWstfL). Draws were sampled using sampling (NUTS). For each parameter, Bulk_ESS and Tail_ESS are effective sample size measures, and Rhat is the potential scale reduction factor on split chains (at convergence, Rhat = 1).

**Group-Level Effects**

| ~spatial_blocks (Number of levels: 120) | | | | | | | |
| --- | --- | --- | --- | --- | --- | --- | --- |
|  | Estimate | Est.Error | l-95% CI | u-95% CI | Rhat | Bulk_ESS | Tail_ESS |
| sd(Intercept) | 0.37 | 0.03 | 0.31 | 0.43 | 1.00 | 801 | 1723 |

**Population-Level Effects**

|  | Estimate | Est.Error | l-95% CI | u-95% CI | Rhat | Bulk_ESS | Tail_ESS |
| --- | --- | --- | --- | --- | --- | --- | --- |
| Intercept | -14.52 | 0.07 | -14.65 | -14.39 | 1.00 | 698 | 1355 |
| climate_classificationTemperate | -0.42 | 0.10 | -0.60 | -0.23 | 1.00 | 651 | 1537 |
| climate_classificationTropical | -0.08 | 0.08 | -0.24 | 0.07 | 1.00 | 707 | 1139 |
| depth_in_m | -0.03 | 0.00 | -0.04 | -0.02 | 1.00 | 3699 | 3248 |

**Family Specific Parameters**

|  | Estimate | Est.Error | l-95% CI | u-95% CI | Rhat | Bulk_ESS | Tail_ESS |
| --- | --- | --- | --- | --- | --- | --- | --- |
| shape | 19.36 | 1.41 | 16.69 | 22.25 | 1.00 | 3433 | 2934 |

**Table S22 | Summary tables of the Bayesian model for the quarter-diurnal 90th percentile of difference in biological rates.** To test whether the quarter-diurnal 90th percentile of difference in biological rates vary significantly between regions (tropical, subtropical and temperate), we implemented a hierarchical modelling approach using Bayesian inference with Stan [(3)](https://www.zotero.org/google-docs/?0KZJr0) and the “brms” package [(4)](https://www.zotero.org/google-docs/?TCpiTY) within the R programming environment [(5)](https://www.zotero.org/google-docs/?yay9co). We specified models by ascribing variation among the data to “climate_classification” and “depth_in_m”, and grouped variation among geographically proximate locations to account for spatial autocorrelation by including a random intercept (“spatial_blocks”) for sampling sites falling within a 174 km radius of each other [(1)](https://www.zotero.org/google-docs/?wSwRhQ). We used an additional level (“plot_id”) of random effects in a crossed design because the time series that were longer than 1 year in duration were divided into 1-year subsamples. Draws were sampled using sampling (NUTS). For each parameter, Bulk_ESS and Tail_ESS are effective sample size measures, and Rhat is the potential scale reduction factor on split chains (at convergence, Rhat = 1).

**Group-Level Effects**

| ~spatial_blocks (Number of levels: 120) | | | | | | | |
| --- | --- | --- | --- | --- | --- | --- | --- |
|  | Estimate | Est.Error | l-95% CI | u-95% CI | Rhat | Bulk_ESS | Tail_ESS |
| sd(Intercept) | 0.47 | 0.05 | 0.37 | 0.57 | 1.00 | 1079 | 1870 |
| ~spatial_blocks:plot_id (Number of levels: 487) | | | | | | | |
|  | Estimate | Est.Error | l-95% CI | u-95% CI | Rhat | Bulk_ESS | Tail_ESS |
| sd(Intercept) | 0.43 | 0.02 | 0.40 | 0.47 | 1.01 | 1025 | 1669 |

**Population-Level Effects**

|  | Estimate | Est.Error | l-95% CI | u-95% CI | Rhat | Bulk_ESS | Tail_ESS |
| --- | --- | --- | --- | --- | --- | --- | --- |
| Intercept | -16.88 | 0.10 | -17.07 | -16.69 | 1.00 | 867 | 1588 |
| climate_classificationTemperate | -0.73 | 0.14 | -0.99 | -0.45 | 1.00 | 1139 | 1997 |
| climate_classificationTropical | 0.07 | 0.12 | -0.15 | 0.29 | 1.00 | 981 | 1888 |
| depth_in_m | -0.03 | 0.01 | -0.05 | -0.01 | 1.00 | 1053 | 1494 |

**Family Specific Parameters**

|  | Estimate | Est.Error | l-95% CI | u-95% CI | Rhat | Bulk_ESS | Tail_ESS |
| --- | --- | --- | --- | --- | --- | --- | --- |
| shape | 10.80 | 0.28 | 10.25 | 11.36 | 1.00 | 4596 | 3055 |

**Table S23 | Summary tables of the Bayesian model for the semi-diurnal 90th percentile of difference in biological rates.** To test whether the semi-diurnal 90th percentile of difference in biological rates vary significantly between regions (tropical, subtropical and temperate), we implemented a hierarchical modelling approach using Bayesian inference with Stan [(3)](https://www.zotero.org/google-docs/?n8NRwR) and the “brms” package [(4)](https://www.zotero.org/google-docs/?Tynu6n) within the R programming environment [(5)](https://www.zotero.org/google-docs/?2gxhCw). We specified models by ascribing variation among the data to “climate_classification” and “depth_in_m”, and grouped variation among geographically proximate locations to account for spatial autocorrelation by including a random intercept (“spatial_blocks”) for sampling sites falling within a 174 km radius of each other [(1)](https://www.zotero.org/google-docs/?8GnJea). We used an additional level (“plot_id”) of random effects in a crossed design because the time series that were longer than 1 year in duration were divided into 1-year subsamples. Draws were sampled using sampling (NUTS). For each parameter, Bulk_ESS and Tail_ESS are effective sample size measures, and Rhat is the potential scale reduction factor on split chains (at convergence, Rhat = 1).

**Group-Level Effects**

| ~spatial_blocks (Number of levels: 120) | | | | | | | |
| --- | --- | --- | --- | --- | --- | --- | --- |
|  | Estimate | Est.Error | l-95% CI | u-95% CI | Rhat | Bulk_ESS | Tail_ESS |
| sd(Intercept) | 0.45 | 0.05 | 0.36 | 0.55 | 1.00 | 1082 | 1935 |
| ~spatial_blocks:plot_id (Number of levels: 487) | | | | | | | |
|  | Estimate | Est.Error | l-95% CI | u-95% CI | Rhat | Bulk_ESS | Tail_ESS |
| sd(Intercept) | 0.42 | 0.02 | 0.39 | 0.46 | 1.00 | 1051 | 1928 |

**Population-Level Effects**

|  | Estimate | Est.Error | l-95% CI | u-95% CI | Rhat | Bulk_ESS | Tail_ESS |
| --- | --- | --- | --- | --- | --- | --- | --- |
| Intercept | -16.45 | 0.10 | -16.65 | -16.26 | 1.01 | 1067 | 1381 |
| climate_classificationTemperate | -0.69 | 0.14 | -0.97 | -0.42 | 1.00 | 1120 | 1759 |
| climate_classificationTropical | 0.05 | 0.11 | -0.17 | 0.28 | 1.00 | 1089 | 1286 |
| depth_in_m | -0.04 | 0.01 | -0.05 | -0.02 | 1.00 | 1066 | 1786 |

**Family Specific Parameters**

|  | Estimate | Est.Error | l-95% CI | u-95% CI | Rhat | Bulk_ESS | Tail_ESS |
| --- | --- | --- | --- | --- | --- | --- | --- |
| shape | 10.86 | 0.29 | 10.30 | 11.44 | 1.00 | 7159 | 3002 |

**Table S24 | Summary tables of the Bayesian model for the diurnal 90th percentile of difference in biological rates.** To test whether the diurnal 90th percentile of difference in biological rates vary significantly between regions (tropical, subtropical and temperate), we implemented a hierarchical modelling approach using Bayesian inference with Stan [(3)](https://www.zotero.org/google-docs/?fOZeWB) and the “brms” package [(4)](https://www.zotero.org/google-docs/?AtJbqc) within the R programming environment [(5)](https://www.zotero.org/google-docs/?PICpSI). We specified models by ascribing variation among the data to “climate_classification” and “depth_in_m”, and grouped variation among geographically proximate locations to account for spatial autocorrelation by including a random intercept (“spatial_blocks”) for sampling sites falling within a 174 km radius of each other [(1)](https://www.zotero.org/google-docs/?nV6mkD). We used an additional level (“plot_id”) of random effects in a crossed design because the time series that were longer than 1 year in duration were divided into 1-year subsamples. Draws were sampled using sampling (NUTS). For each parameter, Bulk_ESS and Tail_ESS are effective sample size measures, and Rhat is the potential scale reduction factor on split chains (at convergence, Rhat = 1).

**Group-Level Effects**

| ~spatial_blocks (Number of levels: 120) | | | | | | | |
| --- | --- | --- | --- | --- | --- | --- | --- |
|  | Estimate | Est.Error | l-95% CI | u-95% CI | Rhat | Bulk_ESS | Tail_ESS |
| sd(Intercept) | 0.43 | 0.05 | 0.34 | 0.52 | 1.00 | 1195 | 2018 |
| ~spatial_blocks:plot_id (Number of levels: 487) | | | | | | | |
|  | Estimate | Est.Error | l-95% CI | u-95% CI | Rhat | Bulk_ESS | Tail_ESS |
| sd(Intercept) | 0.40 | 0.02 | 0.37 | 0.44 | 1.00 | 1054 | 1709 |

**Population-Level Effects**

|  | Estimate | Est.Error | l-95% CI | u-95% CI | Rhat | Bulk_ESS | Tail_ESS |
| --- | --- | --- | --- | --- | --- | --- | --- |
| Intercept | -16.11 | 0.09 | -16.29 | -15.92 | 1.00 | 1624 | 2050 |
| climate_classificationTemperate | -0.65 | 0.13 | -0.92 | -0.39 | 1.00 | 1642 | 2027 |
| climate_classificationTropical | 0.03 | 0.11 | -0.19 | 0.25 | 1.00 | 1714 | 2158 |
| depth_in_m | -0.04 | 0.01 | -0.06 | -0.03 | 1.00 | 1699 | 2246 |

**Family Specific Parameters**

|  | Estimate | Est.Error | l-95% CI | u-95% CI | Rhat | Bulk_ESS | Tail_ESS |
| --- | --- | --- | --- | --- | --- | --- | --- |
| shape | 11.29 | 0.31 | 10.70 | 11.88 | 1.00 | 5398 | 3134 |

**Table S25 | Summary tables of the Bayesian model for the weekly 90th percentile of difference in biological rates.** To test whether the weekly 90th percentile of difference in biological rates vary significantly between regions (tropical, subtropical and temperate), we implemented a hierarchical modelling approach using Bayesian inference with Stan [(3)](https://www.zotero.org/google-docs/?dsPQYC) and the “brms” package [(4)](https://www.zotero.org/google-docs/?htSacv) within the R programming environment [(5)](https://www.zotero.org/google-docs/?ylngb8). We specified models by ascribing variation among the data to “climate_classification” and “depth_in_m”, and grouped variation among geographically proximate locations to account for spatial autocorrelation by including a random intercept (“spatial_blocks”) for sampling sites falling within a 174 km radius of each other [(1)](https://www.zotero.org/google-docs/?bywWm3). We used an additional level (“plot_id”) of random effects in a crossed design because the time series that were longer than 1 year in duration were divided into 1-year subsamples. Draws were sampled using sampling (NUTS). For each parameter, Bulk_ESS and Tail_ESS are effective sample size measures, and Rhat is the potential scale reduction factor on split chains (at convergence, Rhat = 1).

**Group-Level Effects**

| ~spatial_blocks (Number of levels: 120) | | | | | | | |
| --- | --- | --- | --- | --- | --- | --- | --- |
|  | Estimate | Est.Error | l-95% CI | u-95% CI | Rhat | Bulk_ESS | Tail_ESS |
| sd(Intercept) | 0.41 | 0.04 | 0.34 | 0.48 | 1.00 | 1225 | 1478 |
| ~spatial_blocks:plot_id (Number of levels: 487) | | | | | | | |
|  | Estimate | Est.Error | l-95% CI | u-95% CI | Rhat | Bulk_ESS | Tail_ESS |
| sd(Intercept) | 0.30 | 0.01 | 0.27 | 0.33 | 1.00 | 1229 | 2112 |

**Population-Level Effects**

|  | Estimate | Est.Error | l-95% CI | u-95% CI | Rhat | Bulk_ESS | Tail_ESS |
| --- | --- | --- | --- | --- | --- | --- | --- |
| Intercept | -15.50 | 0.08 | -15.66 | -15.35 | 1.00 | 1152 | 1909 |
| climate_classificationTemperate | -0.51 | 0.12 | -0.74 | -0.28 | 1.01 | 1190 | 1811 |
| climate_classificationTropical | 0.03 | 0.10 | -0.16 | 0.22 | 1.00 | 1064 | 1668 |
| depth_in_m | -0.04 | 0.01 | -0.05 | -0.03 | 1.00 | 1303 | 1667 |

**Family Specific Parameters**

|  | Estimate | Est.Error | l-95% CI | u-95% CI | Rhat | Bulk_ESS | Tail_ESS |
| --- | --- | --- | --- | --- | --- | --- | --- |
| shape | 12.83 | 0.35 | 12.15 | 13.50 | 1.00 | 5408 | 2973 |

**Table S26 | Summary tables of the Bayesian model for the bi-weekly 90th percentile of difference in biological rates.** To test whether the bi-weekly 90th percentile of difference in biological rates vary significantly between regions (tropical, subtropical and temperate), we implemented a hierarchical modelling approach using Bayesian inference with Stan [(3)](https://www.zotero.org/google-docs/?mS3dS0) and the “brms” package [(4)](https://www.zotero.org/google-docs/?sflvan) within the R programming environment [(5)](https://www.zotero.org/google-docs/?jx8bhk). We specified models by ascribing variation among the data to “climate_classification” and “depth_in_m”, and grouped variation among geographically proximate locations to account for spatial autocorrelation by including a random intercept (“spatial_blocks”) for sampling sites falling within a 174 km radius of each other [(1)](https://www.zotero.org/google-docs/?Yk2GRC). We used an additional level (“plot_id”) of random effects in a crossed design because the time series that were longer than 1 year in duration were divided into 1-year subsamples. Draws were sampled using sampling (NUTS). For each parameter, Bulk_ESS and Tail_ESS are effective sample size measures, and Rhat is the potential scale reduction factor on split chains (at convergence, Rhat = 1).

**Group-Level Effects**

| ~spatial_blocks (Number of levels: 120) | | | | | | | |
| --- | --- | --- | --- | --- | --- | --- | --- |
|  | Estimate | Est.Error | l-95% CI | u-95% CI | Rhat | Bulk_ESS | Tail_ESS |
| sd(Intercept) | 0.39 | 0.04 | 0.32 | 0.46 | 1.00 | 1143 | 1937 |
| ~spatial_blocks:plot_id (Number of levels: 487) | | | | | | | |
|  | Estimate | Est.Error | l-95% CI | u-95% CI | Rhat | Bulk_ESS | Tail_ESS |
| sd(Intercept) | 0.27 | 0.01 | 0.24 | 0.30 | 1.00 | 1202 | 1842 |

**Population-Level Effects**

|  | Estimate | Est.Error | l-95% CI | u-95% CI | Rhat | Bulk_ESS | Tail_ESS |
| --- | --- | --- | --- | --- | --- | --- | --- |
| Intercept | -15.30 | 0.07 | -15.45 | -15.15 | 1.01 | 1117 | 1674 |
| climate_classificationTemperate | -0.49 | 0.11 | -0.71 | -0.27 | 1.01 | 1348 | 1856 |
| climate_classificationTropical | -0.00 | 0.09 | -0.17 | 0.18 | 1.00 | 1664 | 2370 |
| depth_in_m | -0.04 | 0.01 | -0.05 | -0.03 | 1.00 | 1839 | 2522 |

**Family Specific Parameters**

|  | Estimate | Est.Error | l-95% CI | u-95% CI | Rhat | Bulk_ESS | Tail_ESS |
| --- | --- | --- | --- | --- | --- | --- | --- |
| shape | 13.64 | 0.37 | 12.90 | 14.38 | 1.00 | 4297 | 2841 |

**Table S27 | Summary tables of the Bayesian model for the monthly 90th percentile of difference in biological rates.** To test whether the monthly 90th percentile of difference in biological rates vary significantly between regions (tropical, subtropical and temperate), we implemented a hierarchical modelling approach using Bayesian inference with Stan [(3)](https://www.zotero.org/google-docs/?qUSAWT) and the “brms” package [(4)](https://www.zotero.org/google-docs/?JoDsKZ) within the R programming environment [(5)](https://www.zotero.org/google-docs/?OVYC0G). We specified models by ascribing variation among the data to “climate_classification” and “depth_in_m”, and grouped variation among geographically proximate locations to account for spatial autocorrelation by including a random intercept (“spatial_blocks”) for sampling sites falling within a 174 km radius of each other [(1)](https://www.zotero.org/google-docs/?A6kj1n). We used an additional level (“plot_id”) of random effects in a crossed design because the time series that were longer than 1 year in duration were divided into 1-year subsamples. Draws were sampled using sampling (NUTS). For each parameter, Bulk_ESS and Tail_ESS are effective sample size measures, and Rhat is the potential scale reduction factor on split chains (at convergence, Rhat = 1).

**Group-Level Effects**

| ~spatial_blocks (Number of levels: 120) | | | | | | | |
| --- | --- | --- | --- | --- | --- | --- | --- |
|  | Estimate | Est.Error | l-95% CI | u-95% CI | Rhat | Bulk_ESS | Tail_ESS |
| sd(Intercept) | 0.38 | 0.03 | 0.32 | 0.44 | 1.00 | 1188 | 2054 |
| ~spatial_blocks:plot_id (Number of levels: 487) | | | | | | | |
|  | Estimate | Est.Error | l-95% CI | u-95% CI | Rhat | Bulk_ESS | Tail_ESS |
| sd(Intercept) | 0.23 | 0.01 | 0.21 | 0.25 | 1.00 | 1414 | 2396 |

**Population-Level Effects**

|  | Estimate | Est.Error | l-95% CI | u-95% CI | Rhat | Bulk_ESS | Tail_ESS |
| --- | --- | --- | --- | --- | --- | --- | --- |
| Intercept | -15.12 | 0.07 | -15.25 | -14.98 | 1.00 | 891 | 1511 |
| climate_classificationTemperate | -0.46 | 0.10 | -0.67 | -0.26 | 1.00 | 819 | 1417 |
| climate_classificationTropical | -0.01 | 0.08 | -0.18 | 0.15 | 1.00 | 1084 | 1669 |
| depth_in_m | -0.03 | 0.01 | -0.04 | -0.02 | 1.00 | 1258 | 2036 |

**Family Specific Parameters**

|  | Estimate | Est.Error | l-95% CI | u-95% CI | Rhat | Bulk_ESS | Tail_ESS |
| --- | --- | --- | --- | --- | --- | --- | --- |
| shape | 14.06 | 0.40 | 13.29 | 14.85 | 1.00 | 5050 | 2680 |

**Table S28 | Summary tables of the Bayesian model for the annual 90th percentile of difference in biological rates.** To test whether the annual 90th percentile of difference in biological rates vary significantly between regions (tropical, subtropical and temperate), we implemented a hierarchical modelling approach using Bayesian inference with Stan [(3)](https://www.zotero.org/google-docs/?QkoSfW) and the “brms” package [(4)](https://www.zotero.org/google-docs/?g0eVge) within the R programming environment [(5)](https://www.zotero.org/google-docs/?iBVWn9). We specified models by ascribing variation among the data to “climate_classification” and “depth_in_m”, and grouped variation among geographically proximate locations to account for spatial autocorrelation by including a random intercept (“spatial_blocks”) for sampling sites falling within a 174 km radius of each other [(1)](https://www.zotero.org/google-docs/?Lq4MaW). Draws were sampled using sampling (NUTS). For each parameter, Bulk_ESS and Tail_ESS are effective sample size measures, and Rhat is the potential scale reduction factor on split chains (at convergence, Rhat = 1).

**Group-Level Effects**

| ~spatial_blocks (Number of levels: 120) | | | | | | | |
| --- | --- | --- | --- | --- | --- | --- | --- |
|  | Estimate | Est.Error | l-95% CI | u-95% CI | Rhat | Bulk_ESS | Tail_ESS |
| sd(Intercept) | 0.36 | 0.03 | 0.30 | 0.42 | 1.01 | 889 | 1494 |

**Population-Level Effects**

|  | Estimate | Est.Error | l-95% CI | u-95% CI | Rhat | Bulk_ESS | Tail_ESS |
| --- | --- | --- | --- | --- | --- | --- | --- |
| Intercept | -14.42 | 0.06 | -14.54 | -14.29 | 1.00 | 752 | 1184 |
| climate_classificationTemperate | -0.37 | 0.10 | -0.57 | -0.19 | 1.00 | 549 | 1005 |
| climate_classificationTropical | -0.03 | 0.08 | -0.18 | 0.13 | 1.00 | 803 | 1300 |
| depth_in_m | -0.02 | 0.00 | -0.03 | -0.02 | 1.00 | 4292 | 2995 |

**Family Specific Parameters**

|  | Estimate | Est.Error | l-95% CI | u-95% CI | Rhat | Bulk_ESS | Tail_ESS |
| --- | --- | --- | --- | --- | --- | --- | --- |
| shape | 22.13 | 1.61 | 19.03 | 25.39 | 1.00 | 2780 | 2914 |

**Table S29 | Breakdown of time series from tropical regions.**

| **Data source** | **Number of time series** |
| --- | --- |
| The Australian Institute of Marine Science | 66 |
| Pacific Marine Environmental Laboratory | 38 |
| Maria Azeredo de Dornelas | 21 |
| Brian Helmuth et al. | 19 |
| Julia Baum | 9 |
| Sarah Davies | 5 |
| Guilherme Longo | 4 |
| Alex S.J. Wyatt | 3 |
| Hydrographic and Oceanographic Service of the Chilean Navy | 2 |
| John Bruno | 1 |
| SiMCosta | 1 |

**Table S30 | Main temperature loggers used by each data provider, with information pertaining to their accuracy and precision, where reported.** In cases where multiple loggers were used, only the lowest accuracy and precision are reported (to be conservative). Although information on the accuracy and precision could not be obtained in some cases, we do not expect this to have any significant impact on our overall results (see Results - Potential limitations).

| **Data source** | **Number of sites** | **Accuracy**  **(°C)** | **Precision (°C)** | **Loggers used** | **Other notes** |
| --- | --- | --- | --- | --- | --- |
| The Australian Institute of Marine Science | 109 | 0.8 | 0.01 | Sensus Ultra loggers (produced by ReefNet Inc., Canada), VEMCO Minilog-II-T loggers | Sensors were changed over time. The lower accuracy and precision are recorded here. |
| Santa Barbara Coastal LTER | 49 | 0.21 | 0.02 | Hobo UTBI-001 |  |
| Zachary L. Monteith | 48 | 0.53 | 0.14 | HOBO Tidbit v2 Temp data loggers and HOBO Pendant Temperature/Light data loggers | 2 different loggers were used. The lower accuracy and precision are recorded here. |
| Pacific Marine Environmental Laboratory | 38 | 0.03 | 0.001 | See:  https://www.pmel.noaa.gov/gtmba/sensor-specifications | Sensors were changed over time. The lowest accuracy and precision are recorded here. More detail: https://www.pmel.noaa.gov/gtmba/sensor-specifications |
| Carlo Caruso | 30 | 0.53 | 0.14 | HOBO Pendant Temperature/Light 64K Data Logger | In the metadata file, Elizabeth Madin is listed as data provider, but it was Carlo Caruso who collected the data. |
| Hydrographic and Oceanographic Service of the Chilean Navy | 29 | No info | No info | No info | Information on loggers could not be obtained |
| COSYNA | 28 | 0.3 | 0.1 | Seabird SBE37 and Sea and Sun Technology T40 and PT-100 | Different loggers were used. The lowest accuracy and precision are recorded here.  Data provider noted that due to biofouling, sensor drifts up to 0.03 °C were observed for some of the time series between the two to three weekly instrument cleansings on a pole device with more instrumentation that allowed the quantification of this phenomenon. |
| Maria Azeredo de Dornelas | 21 | 0.53 | 0.14 | HOBO Pendant Temperature/Light 64K Data Logger |  |
| Helmuth Brian | 19 | 0.2 | 0.02 | HOBO Loggers |  |
| Garrabou Joaquim | 16 | 0.2 | 0.02 | HOBO Loggers |  |
| Integrated Ocean Observing System | 15 | 0.005 | 0.001 | Sea-Bird SBE37 |  |
| IMAS Data Portal | 12 | 0.21 | 0.02 | HOBO v2 Water Temp Pro sensor, Onset |  |
| SiMCosta | 12 | 0.002 | 0.001 | SeaBird WQMx sensors and SeaBird MicroCAT sensors | 2 different loggers were used. The lower accuracy and precision are recorded here. |
| Alberto Lindner | 11 | 0.53 | 0.14 | HOBO Pendant® Temperature Data Logger UA-002 |  |
| Julia Baum | 9 | 0.002 | 0.0001 | Seabird 56s |  |
| Centro de Datos Oceanograficos y Meteorologicos | 7 | No info | No info | No info | Information on loggers could not be obtained |
| Alex S.J. Wyatt | 6 | 0.002 | 0.0001 | SeaBird SBE 56 |  |
| Jennifer Jackson | 6 | 0.21 | 0.02 | SBE 16plus V2 and HOBO TidbiT v2 Water Temperature Data Logger | 2 different loggers were used. The lower accuracy and precision are recorded here. |
| Davies Sarah | 5 | 0.21 | 0.02 | HOBO v2 Water Temp Pro sensor, Onset |  |
| Guilherme Longo | 4 | 0.5 | 0.04 | HOBO Loggers |  |
| Southern African Data Centre for Oceanography | 3 | 0.05 | No info | No info | Accuracy detail found here (DEA collection): https://journals.plos.org/plosone/article?id=10.1371/journal.pone.0081944.  Precision details cannot be found |
| Brazil's Navy | 2 | 0.1 | 0.01 | Signature500 ADCP |  |
| British Oceanographic Data Centre | 2 | 0.002 | 0.0001 | Sea-Bird SBE 37 MicroCat IMP-CT |  |
| David Kushner | 2 | No info | 0.2 | Onset Inc. temperature loggers | Some uncertainties in the values due to change of loggers over time |
| ISRAMAR | 2 | 0.005 | 0.0001 | SBE 19Plus V2 |  |
| Bodega Ocean Observing Node | 1 | 0.005 | 0.0001 | Sea-Bird Electronics SBE 16+ SEACAT |  |
| Bruno John | 1 | 0.21 | 0.02 | HOBO v2 Water Temp Pro sensor, Onset |  |
| Chris Neufeld | 1 | 0.2 | 0.1 | ENVloggers: https://electricblue.eu/envloggers |  |
| Dong Yunwei | 1 | 0.002 | 0.0001 | HydroCAT-EP v2 |  |
| Gagnon Patrick | 1 | 0.53 | 0.14 | HOBO Pendant Temperature/Light 64K Data Logger |  |
| Kersting Diego | 1 | 0.21 | 0.02 | HOBO v2 Water Temp Pro sensor, Onset |  |
| Western Channel Observatory | 1 | 0.005 | 0.0001 | SBE 19Plus V2 |  |

**SI References**

1. [K. Hosoda, H. Kawamura, Global space-time statistics of sea surface temperature estimated from AMSR-E data. *Geophys. Res. Lett.* **31** (2004).](https://www.zotero.org/google-docs/?Xa94Jt)
2. [R. V. Lenth, *et al.*, emmeans: Estimated Marginal Means, aka Least-Squares Means (2022) (October 20, 2022).](https://www.zotero.org/google-docs/?Xa94Jt)
3. [B. Carpenter, *et al.*, Stan: A Probabilistic Programming Language. *J. Stat. Softw.* **76**, 1–32 (2017).](https://www.zotero.org/google-docs/?Xa94Jt)
4. [P.-C. Bürkner, brms: An R Package for Bayesian Multilevel Models Using Stan. *J. Stat. Softw.* **80**, 1–28 (2017).](https://www.zotero.org/google-docs/?Xa94Jt)
5. [R Core Team, R: A language and environment for statistical computing. (2020).](https://www.zotero.org/google-docs/?Xa94Jt)

**Additional supporting references (for data and *R* packages)**

70. D. Li Shing Hiung, Data (2022) https:/doi.org/10.6084/m9.figshare.21386430.v5 (November 30, 2023).

71. re3data.org: COSYNA Data web portal; editing status 2023-05-15; re3data.org - Registry of Research Data Repositories. http://doi.org/10.17616/R3K02T last accessed: 2024-01-28

72. Faria-Junior Edson, Alberto Lindner (2019). An underwater temperature dataset from coastal islands in Santa Catarina, southern Brazil: high accuracy data from different depths. SEANOE. <https://doi.org/10.17882/62120>

73. Australian Institute of Marine Science (AIMS). (2017). AIMS Sea Water Temperature Observing System (AIMS Temperature Logger Program). <https://doi.org/10.25845/5b4eb0f9bb848>, date accessed {05-05-2021}

74. Santa Barbara Coastal LTER, L. Washburn, C. Gotschalk, and D. Salazar. 2021. SBC LTER: Ocean: Currents and Biogeochemistry: Moored CTD and ADCP data from Alegria Reef Mooring (ALE), ongoing since 1999 ver 13. Environmental Data Initiative. https://doi.org/10.6073/pasta/982ae2643c80dd5b64aedca03da24ecb (Accessed 2024-01-29).

75. Santa Barbara Coastal LTER, L. Washburn, C. Gotschalk, and D. Salazar. 2021. SBC LTER: Ocean: Currents and Biogeochemistry: Moored CTD and ADCP data from Arroyo Quemado Reef Mooring (ARQ), ongoing since 2004 ver 23. Environmental Data Initiative. https://doi.org/10.6073/pasta/b463b1f3e1fdadcf706650d5bd1530e1 (Accessed 2024-01-29).

76. Santa Barbara Coastal LTER, L. Washburn, C. Gotschalk, and D. Salazar. 2021. SBC LTER: Ocean: Currents and Biogeochemistry: Moored CTD and ADCP data from Naples Reef Mooring (NAP), ongoing since 2001 ver 29. Environmental Data Initiative. https://doi.org/10.6073/pasta/29b41358edfe822185707b07f7b6ced3 (Accessed 2024-01-29).

77. Santa Barbara Coastal LTER, L. Washburn, C. Gotschalk, and D. Salazar. 2021. SBC LTER: Ocean: Currents and Biogeochemistry: Moored CTD and ADCP data from Mohawk Outside Spar (MKO), ongoing since 2005 ver 15. Environmental Data Initiative. https://doi.org/10.6073/pasta/6dec3fe25b16469012c5337b546c4dfa (Accessed 2024-01-29).

78. Santa Barbara Coastal LTER, L. Washburn, C. Gotschalk, and D. Salazar. 2021. SBC LTER: Ocean: Currents and Biogeochemistry: Moored CTD and ADCP data from Carpinteria Reef Mooring (CAR, ongoing since 2001 ver 25. Environmental Data Initiative. https://doi.org/10.6073/pasta/85084765545abe3ab284c984b89448e8 (Accessed 2024-01-29).

79. Santa Barbara Coastal LTER, L. Washburn, C. Gotschalk, and D. Salazar. 2021. SBC LTER: Ocean: Currents and Biogeochemistry: Moored CTD and ADCP data from Santa Barbara Harbor Mooring (SBH), ongoing since 1999 ver 4. Environmental Data Initiative. https://doi.org/10.6073/pasta/0faed93009eff16a61d90467170eab7d (Accessed 2024-01-29).

80. Santa Barbara Coastal LTER, L. Washburn, and L. Kui. 2021. SBC LTER: Ocean: Ocean hourly temperature at nearshore locations along the Northern Channel Islands in the Santa Barbara Channel, ongoing since 2000 ver 1. Environmental Data Initiative. https://doi.org/10.6073/pasta/1dfe35ec63376ba1f02172ab92f8032e (Accessed 2024-01-29).

81. Santa Barbara Coastal LTER and L. Washburn. 2014. SBC LTER: Ocean: Ocean Currents and Biogeochemistry: Moored CTD and ADCP data from Arroyo Quemado, Site AQM ver 15. Environmental Data Initiative. https://doi.org/10.6073/pasta/b33b99531b3210d17d9844706ba929d0 (Accessed 2024-01-29).

82. Santa Barbara Coastal LTER and L. Washburn. 2014. SBC LTER: Ocean: Currents and Biogeochemistry: Moored CTD and ADCP data from Arroyo Burro Reef Mooring (ARB) ver 11. Environmental Data Initiative. https://doi.org/10.6073/pasta/b43b1c5042192dade8927967a986561b (Accessed 2024-01-29).

83. D. Kahle and H. Wickham. ggmap: Spatial Visualization with ggplot2. The R Journal, 5(1), 144-161. URL http://journal.r-project.org/archive/2013-1/kahle-wickham.pdf

84. Hadley Wickham (2011). The Split-Apply-Combine Strategy for Data Analysis. Journal of Statistical Software, 40(1), 1-29. URL <https://www.jstatsoft.org/v40/i01/>

85. Baschek et al: The Coastal Observing System for Northern and Arctic Seas (COSYNA), Ocean Sci., 13, 379–410, <https://doi.org/10.5194/os-13-379-2017>

86. Breitbach et al: Accessing diverse data comprehensively – CODM, the COSYNA data portal, Ocean Sci., 12, 909–923, <https://doi.org/10.5194/os-12-909-2016>

88. BRAZIL HYDROGRAPHY CENTER - CHM, 2021, available at <https://www.marinha.mil.br/chm/dados-do-goos-brasil/pnboia-mapa> accessed on July 2021

89. Wickham H, François R, Henry L, Müller K, Vaughan D (2023). _dplyr: A Grammar of

Data Manipulation_. R package version 1.1.4,

<https://CRAN.R-project.org/package=dplyr>.

90. Auguie B (2017). _gridExtra: Miscellaneous Functions for "Grid" Graphics_. R

package version 2.3, <https://CRAN.R-project.org/package=gridExtra>.

91. Kassambara A (2023). _ggpubr: 'ggplot2' Based Publication Ready Plots_. R package

version 0.6.0, <https://CRAN.R-project.org/package=ggpubr>.

92. Garcia de la Garza A, Vandekar S, Roalf D, Ruparel K, Gur R, Gur R, Satterthwaite

T, Shinohara R (2018). _voxel: Mass-Univariate Voxelwise Analysis of Medical

Imaging Data_. R package version 1.3.5,

<https://CRAN.R-project.org/package=voxel>.

93. Wickham H, Pedersen T, Seidel D (2023). _scales: Scale Functions for

Visualization_. R package version 1.3.0,

<https://CRAN.R-project.org/package=scales>.

94. Grothendieck G (2018). _gsubfn: Utilities for Strings and Function Arguments_. R

package version 0.7, <https://CRAN.R-project.org/package=gsubfn>.

95. Becker OScbRA, Minka ARWRvbRBEbTP, Deckmyn. A (2023). _maps: Draw Geographical

Maps_. R package version 3.4.2, <https://CRAN.R-project.org/package=maps>.

96. Wickham H, Averick M, Bryan J, Chang W, McGowan LD, François R, Grolemund G, Hayes A, Henry L, Hester J, Kuhn M, Pedersen TL, Miller E, Bache SM, Müller K, Ooms J,

Robinson D, Seidel DP, Spinu V, Takahashi K, Vaughan D, Wilke C, Woo K, Yutani H

(2019). “Welcome to the tidyverse.” _Journal of Open Source Software_, *4*(43),

1686. doi:10.21105/joss.01686 <https://doi.org/10.21105/joss.01686>.

97. Pierce D (2023). _ncdf4: Interface to Unidata netCDF (Version 4 or Earlier) Format

Data Files_. R package version 1.22, <https://CRAN.R-project.org/package=ncdf4>.

98. Kay M (2023). _tidybayes: Tidy Data and Geoms for Bayesian Models_.

doi:10.5281/zenodo.1308151 <https://doi.org/10.5281/zenodo.1308151>, R package

version 3.0.6, <http://mjskay.github.io/tidybayes/>.

99. Tiedemann F (2022). _gghalves: Compose Half-Half Plots Using Your Favourite

Geoms_. R package version 0.1.4, <https://CRAN.R-project.org/package=gghalves>.

100. Clarke E, Sherrill-Mix S, Dawson C (2023). _ggbeeswarm: Categorical Scatter

(Violin Point) Plots_. R package version 0.7.2,

<https://CRAN.R-project.org/package=ggbeeswarm>.
